# CD33 and SHP-1/*PTPN6* Interaction in Alzheimer’s Disease

**DOI:** 10.3390/genes15091204

**Published:** 2024-09-13

**Authors:** Lien Beckers, Mamunur Rashid, Annie J. Lee, Zena K. Chatila, Kirstin A. Tamucci, Ryan C. Talcoff, Jennifer L. Hall, David A. Bennett, Badri N. Vardarajan, Elizabeth M. Bradshaw

**Affiliations:** 1 Department of Neurology, Columbia University Irving Medical Center, New York, NY 10032, USA; lien.beckers@hotmail.com (L.B.); mr3983@cumc.columbia.edu (M.R.); ajl2199@cumc.columbia.edu (A.J.L.); zkc2001@cumc.columbia.edu (Z.K.C.); kat2177@cumc.columbia.edu (K.A.T.); rt2857@cumc.columbia.edu (R.C.T.); jlg2267@cumc.columbia.edu (J.L.H.); bnv2103@cumc.columbia.edu (B.N.V.); 2 Division of Translational Neurobiology, Department of Neurology, Columbia University Irving Medical Center, New York, NY 10032, USA; 3 The Carol and Gene Ludwig Center for Research on Neurodegeneration, Columbia University Irving Medical Center, New York, NY 10032, USA; 4 Gertrude H. Sergievsky Center, College of Physicians and Surgeons, Columbia University, New York, NY 10032, USA; 5 The Taub Institute for Research on Alzheimer’s Disease and the Aging Brain, New York, NY 10032, USA; 6 Rush Alzheimer Disease Center, Rush University Medical Center, Chicago, IL 60612, USA; david_a_bennett@rush.edu

**Keywords:** Alzheimer’s disease, microglia, CD33, SHP-1

## Abstract

Large-scale genetic studies have identified numerous genetic risk factors that suggest a central role for innate immune cells in susceptibility to Alzheimer’s disease (AD). CD33, an immunomodulatory transmembrane sialic acid binding protein expressed on myeloid cells, was identified as one such genetic risk factor associated with Alzheimer’s disease. Several studies explored the molecular outcomes of genetic variation at the *CD33* locus. It has been determined that the risk variant associated with AD increases the expression of the large isoform of CD33 (CD33M) in innate immune cells and alters its biological functions. CD33 is thought to signal via the interaction of its ITIM motif and the protein tyrosine phosphatase, SHP-1. Here, we utilize different molecular and computational approaches to investigate how AD-associated genetic variation in CD33 affects its interaction with SHP-1 in human microglia and microglia-like cells. Our findings demonstrate a genotype-dependent interaction between CD33 and SHP-1, which may functionally contribute to the AD risk associated with this *CD33* variant. We also found that *CD33-PTPN6* (SHP-1) gene–gene interactions impact AD-related traits, while *CD33-PTPN11* (SHP-2) interactions do not.

## 1. Introduction

Alzheimer’s disease (AD) is an age-related neurodegenerative disease and the most common form of dementia, characterized initially by short-term memory loss and disorientation, followed by progressive memory loss and cognitive decline [1]. Classical neuropathological features of AD include presymptomatic accumulation of extracellular amyloid β (Aβ) aggregates, which form senile plaques, intracellular aggregation of hyperphosphorylated tau protein, causing neurofibrillary tangles (NFTs), and extensive neuronal loss [2,3]. AD has become an urgent global health problem, with an enormous socioeconomic burden. The prevention and treatment of cognitive decline due to AD is a public health priority. However, AD has vast heterogeneity clinically, cellularly, and molecularly, illustrated by resilience to neuropathology, the presence of comorbidities, and genetic background, complicating therapeutic efforts [4]. Thus, there is a pressing need for therapeutics that modify the disease course and reverse the underlying pathology with a more personalized medicine approach. Genetics is one tool we can use to develop personalized targeted therapies.

With the discovery and validation of AD susceptibility loci, we now have more than 70 AD risk factors that give insights into the earliest steps in the pathophysiological processes leading to AD [5,6]. These genome-wide association and sequencing studies have identified and validated several genes, including *CD33*, that are associated with AD susceptibility and have implicated the innate immune system in disease onset [7,8,9,10,11,12,13]. In the case of *CD33*, the *CD33* rs3865444^C^ risk allele results in increased surface density of full-length CD33 protein on myeloid cells (monocytes and microglia), as well as a reduced ability to phagocytose amyloid β, leading to accumulation of neuritic amyloid plaques [14]. Further, we have demonstrated that this risk allele leads to greater accumulation of amyloid both in asymptomatic individuals using in vivo amyloid imaging and in post-mortem brain tissue using a detailed quantitation of amyloid pathology [14].

CD33 is also known as Siglec-3 and is a 67 KDa transmembrane glycoprotein expressed on the surface of myeloid cells. It functions as a sialic acid binding receptor and is an inhibitory signaling molecule: its intracellular component contains putative immunoreceptor tyrosine-based inhibitory motifs (ITIMs) that are implicated in the inhibition of cellular activity. The *CD33* risk variant leads to decreased splicing of exon 2, which contains the sialic acid binding domain. This splicing suggests that the function of CD33 binding to sialic acid and bringing signaling receptors into close proximity to dampen the function of these receptors is critical for AD risk [15]. Understanding how CD33 exerts its downstream effect on signaling proteins in microglia is crucial for our long-term goal of determining whether CD33 or its binding partners are tractable therapeutic targets for AD.

SHP-1 (encoded by *PTPN6*) is a protein tyrosine phosphatase that has been functionally linked to the inhibitory effects of CD33 signaling; it was found to bind to the ITIM motif of CD33 in both the THP1 and U937 myeloid cell lines after pervanadate treatment, which inhibits tyrosine phosphatases [16]. SHP-2 (encoded by *PTPN11*) is a homolog of SHP-1 and shares significant structural and functional similarities with SHP-1, including CD33 binding. While both SHP-1 and SHP-2 can bind to ITIM motifs and regulate signaling pathways, SHP-1 is thought to have a higher affinity for CD33 [16,17]. CD33 knockdown and/or SHP-1 reduction resulted in a shared phenotype of increased SYK phosphorylation and ERK1/2 signaling, inflammatory gene transcription, and phagocytosis of amyloid β in the human macrophage THP1 cell line and human iPSC microglia [18]. These findings have identified SHP-1 as a potential target to modulate immune activation in AD. However, the influence of the CD33/SHP-1 interaction on the association between CD33 and AD has yet to be explored.

We hypothesize that CD33 and SHP-1 are binding partners and that their interaction in microglia participates in AD risk. In this work, we demonstrate that CD33 and SHP-1 are binding partners in human microglia-like cells in vitro and in human microglia in situ in post-mortem brain tissue. This interaction is increased in a phosphorylation-state and genotype-specific manner in microglia-like cells of the *CD33* AD risk variant rs3865444^CC^. We further demonstrate that there are interactions between *CD33* gene expression and *PTPN6* gene expression on amyloid, tangles, pathologic AD, and global AD pathology burden.

## 2. Materials and Methods

### 2.1. Isolation and Differentiation of Human Monocytes into Monocyte-Derived Microglia-like Cells (MDMi)

Peripheral blood mononuclear cells (PBMCs) were extracted from blood samples from the New York Blood Center (NYBC) cohort using a standard Ficoll protocol (Ficoll-Paque TM PLUS) with Lymphoprep gradient centrifugation (StemCell Technologies, Vancouver, BC, Canada). PBMCs were frozen at a concentration of 1 × 10^7^ to 3 × 10^7^ cells/mL in 10% dimethyl sulfoxide (DMSO; Sigma-Aldrich, St. Louis, MO, USA) and 90% (*v*/*v*) fetal bovine serum (FBS; Corning, Tarboro, NC, USA). Frozen PBMCs were thawed and washed in 10 mL of phosphate-buffered saline (PBS). CD14^+^ monocytes were purified from whole PBMCs using anti-CD14^+^ microbeads (Miltenyi Biotec, Gaithersburg, MD, USA) and plated at 1.5 × 10^5^ cells in a 96-well plate. To induce the differentiation of MDMi, monocytes were cultured in RPMI-1640 Glutamax (Life Technologies, Greencastle, PA, USA) supplemented with 1% P/S (Lonza, Basel, Switzerland), Fungizone (2.5 μg/mL; Life Technologies), and a mixture of the following human recombinant cytokines: macrophage colony-stimulating factor (M-CSF; 10 ng/mL; BioLegend, San Diego, CA, USA, 574806), granulocyte-macrophage colony-stimulating factor (GM-CSF; 10 ng/mL; R&D Systems, Minneapolis, MN, USA, 215-GM-010/CF), nerve growth factor–β (NGF-β; 10 ng/mL; R&D Systems, 256-GF-100), chemokine ligand 2 (CCL2; 100 ng/mL; BioLegend, San Diego, CA, USA. 571404), and Interleukin-34 (IL-34; 100 ng/mL; R&D Systems, 5265-IL-010/CF) under standard humidified culture conditions (37 °C, 5% CO_2_) for 10 days [19]. Since our initial publication, this model has been adopted by many groups and has been consistently shown to be a valid model of human microglia to study neurodegenerative disease [20,21,22,23].

### 2.2. Tyrosine Phosphatase Inhibitor Treatment

MDMi were treated with the protein tyrosine phosphatase inhibitor sodium orthovanadate (pervanadate) immediately before performing proximity ligation assays as described below. A stock solution of 1 mM sodium orthovanadate was freshly made with 3% hydrogen peroxide (H_2_O_2_) in water. After catalase was added, 100 μM sodium orthovanadate in media was added to the cells and incubated for 15 min at 37 °C. The untreated control cells were incubated with media without pervanadate.

### 2.3. Proximity Ligation Assay of MDMi and Human Brain Tissue

MDMi and frozen tissue sections were prepared as described below and incubated with the following primary antibodies: Primary antibodies used were mouse anti-SHP-1 (Thermo Fisher, Fair Lawn, NJ, USA, Cat# MA5-11669) and rabbit anti-CD33 (Sigma, St. Louis, MO, USA, Cat# HPA035832).

MDMi (cells): MDMi were stained in flat-bottom 96-well plates (corning costar #3603). After 10 days of incubation in RPMI-1640 Glutamax supplemented with 1% P/S, Fungizone (2.5 μg/mL), and a mixture of human cytokines (described above), MDMi were centrifuged at 1200 rpm for 5 min. Cells were blocked with Fc receptor blocking solution (Human TruStain FcX, Biolegend, San Diego, CA, USA, 1:20) for 10 min on ice. After centrifugation at 1200 rpm for 5 min, MDMi were fixed using BD Cytofix/CytoPerm (Thermo Fisher, Fair Lawn, NJ, USA,) for 20 min on ice. Cells were then washed with BD Perm/Wash (Thermo Fisher, Fair Lawn, NJ, USA,) and blocked with PBS containing 5% normal goat serum (Thermo Fisher, Fair Lawn, NJ, USA, 31872), and 2% bovine serum albumin (BSA) in PBS. Cells were then incubated with primary antibodies overnight.

Human tissues: Frozen sections of human prefrontal cortex were fixed in 100% ethanol for 15 min at −20 °C after thawing them for 10 min. Slides were washed three times with 1× PBS. The sections were then blocked with 3% BSA in 1× PBS containing 0.1% Triton-X for 1 h at room temperature. Tissues were then incubated with primary antibodies overnight.

Following overnight incubation with primary antibodies, a humid chamber with water was heated to 37 °C. All procedures were performed at 37 °C. The cells or tissue sections were washed three times with PBS and subsequently incubated with a plus and minus probe (Sigma, St. Louis, MO, USA; Probe Duolink PLA rabbit PLUS, DUO92002; Probe Duolink PLA mouse MINUS, DUO92004; Probe Duolink PLA mouse PLUS, DUO92001; Probe Duolink PLA goat MINUS, DUO92006; 150 µL/tissue section, 80 µL/well for cells) dissolved in 1× Antibody Diluent buffer (Sigma, St. Louis, MO, USA, provided with kit) for 1.5 h (tissue sections) or on a shaker (60 rpm) for 1 h (cells). After one (tissue) or two (cells) washes for 5 min with Duolink In Situ Wash Buffer A (Sigma, St. Louis, MO, USA, DUO82049), Duolink Ligation mixture (Sigma, St. Louis, MO, USA, DUO92008) was added for 1.5 h (tissue, 150 µL/section) or 30–40 min on rotor (cells, 80 µL/well). Samples were washed once (tissue) or twice (cells) for 2 min with Wash Buffer A, and then incubated with Duolink Amplification mixture (Sigma, St. Louis, MO, USA, DUO92008) for 1.5 h in dark (tissue, 150 µL/section; cells, 80 µL/well, on 60 rpm rotator). Then, samples were washed with Duolink In Situ Wash Buffer B (Sigma, St. Louis, MO, USA, DUO82049) for 2 min once (tissue) or twice (cells). Subsequently, Duolink mounting medium with DAPI (Sigma, St. Louis, MO, USA, DUO82040) was added to cells (80 µL/well). After washing with Wash Buffer B, tissue sections were covered with 0.3% Sudan Black for 10 min at room temperature, then washed with PBS, and finally mounted with two drops of Duolink mounting medium with DAPI.

Fluorescence was acquired with a confocal laser scanning microscope (LSM 700, Zeiss, Oberkochen, Germany) and a Zeiss Axio Observer Z1 fluorescence microscope, running Zen 2012 SP2 software (Zeiss, Oberkochen, Germany). Images were exported to and quantified by using Image J2 software (Version 2.14.0/1.54f, NIH, Bethesda, MD, USA). PLA interactions were quantified as PLA dots per live DAPI+ nucleated cell.

### 2.4. Genotyping

For genotyping of the blood or brain samples, DNA was isolated with the PureLink Mini Kit (Thermo Fisher, Fair Lawn, NJ, USA,) and quantified by nanodrop. Once the DNA was isolated, it was diluted to 25 ng/µL. Genotyping for the *CD33* SNP rs3865444 was performed with the C_1487395_40 assay (Thermo Fisher, Fair Lawn, NJ, USA,).

### 2.5. Statistical Analysis

Data are presented as the mean ± SEM. A comparison of two groups was performed using an unpaired *t*-test. For multiple-group comparisons, analysis of variance (ANOVA) with post hoc Tukey correction was applied. Statistical analyses were performed using GraphPad Prism 5.0 (GraphPad Software Inc., San Diego, CA, USA). A *p*-value of <0.05 was considered significant.

### 2.6. Gene–Gene Interactions

Gene–gene interactions were investigated using the ROSMAP RNA-sequencing and GWAS data that have been previously described to examine the interplay between *CD33* and *PTPN6* using three models [24,25]. All ROSMAP participants were enrolled without known dementia and agreed to detailed clinical evaluation and brain donation at death [26]. Clinical and pathologic methods have been previously described [27,28,29,30,31,32]. Linear regression models for quantitative traits and logistic regression models for binary traits were used to test the association of *CD33* gene expression with *PTPN6* levels in the dorsolateral prefrontal cortex (DLPFC) with (a) neuropathological LOAD status, (b) β-amyloid levels, (c) neurofibrillary tangle (NFT) burden, (d) global measure of pathology based on the scaled scores of 5 brain regions, (e) estimated slope of global cognition using longitudinal measurements, (f) hippocampal sclerosis, (g) TDP43 pathology and (h) clinical dementia diagnosis antemortem. The gene expression levels were adjusted for age, sex, RIN score, post-mortem interval, and other technical covariates for RNA-sequencing and the residual expression values were used for interaction testing. A quantitative trait loci approach was used to test whether genetic variation in *PTPN6* regulates *CD33* expression levels and vice versa. Common SNPs (MAF > 0.01) were tested for association with residual gene expression levels in a linear regression model adjusting for age, sex, and population substructure variables. Lastly, the R mediation package was used to test whether the association of *CD33* expression with clinical AD, AD neuropathology and cognition is mediated by *PTPN6* levels. This package was also used to test mediation in the other direction (*CD33* mediates *PTPN6* association with traits) to establish causality. This modeling was repeated to examine the interplay between *CD33* and *PTPN11* (gene for SHP-2).

## 3. Results

### 3.1. CD33 and SHP-1 Interact in Human Microglia-like Cells in a CD33 Genotype-Sensitive Manner

**Figure 1:**
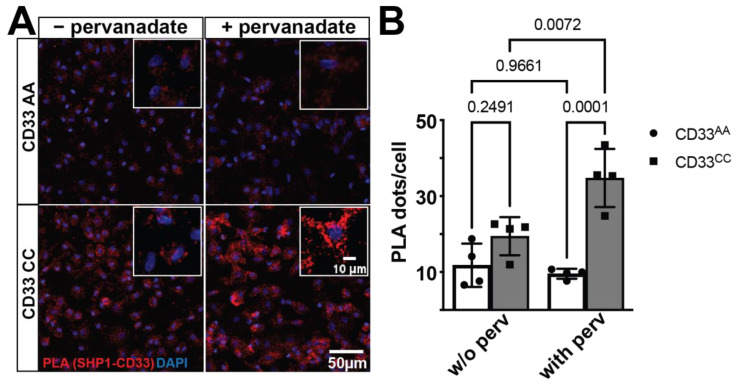
CD33 and SHP-1 interact in a genotype-dependent manner. (**A**) A representative confocal image of MDMi cells stained for proximity ligation assays (PLA). The proximity ligation puncta (red dots, zoomed in inset) represent the protein–protein interaction between CD33 and SHP-1. DAPI (blue) is used to counterstain the nucleus. (**B**) Quantification of the PLA dots shows a significant increase in the PLA counts in the CD33 CC group (*n* = 4) when the cells are treated with pervanadate compared to the CD33 AA group (*n* = 4). Data are represented as the mean ± SEM. Two-way ANOVA followed by Tukey’s multiple comparisons test. Scale bar: 50 μm; 10 μm inset.

To investigate the interaction between CD33 and SHP-1, proximity ligation assay (PLA) was performed on primary monocyte-derived microglia-like (MDMi) cell cultures from individuals genotyped for the *CD33* AD-associated variant, rs3865444. As PLA detects protein–protein interactions in situ at a distance <40 nm, we used this assay to measure the number of interactions per cell [33]. The *CD33* AD-associated risk variant, rs3865444^CC^, increases CD33 surface expression on human monocytes, microglia, and MDMi [14,19]. We confirm that CD33 protein expression is increased in MDMi from rs3865444^CC^ individuals, and we found no genotype effect on SHP-1 protein expression (Supplemental Figure S1). Here, we demonstrate that CD33 and SHP-1 interact in the PLA assay (Figure 1). However, the number of PLA dots, which represent singular interactions, does not differ between genotypes at the baseline level. As SHP-1 is a phosphatase, we next investigated whether this interaction is sensitive to the phosphorylation state. We treated MDMi with pervanadate, which inhibits phosphatase activity, resulting in globally increased phosphorylation [34]. PLA demonstrated increased CD33-SHP-1 interaction in the context of pervanadate but only in MDMi from individuals homozygous for the risk allele, suggesting that this interaction is both sensitive to the phosphorylation state and to the genetic background of the individual. In the presence of pervanadate, there was a significant increase in the number of CD33-SHP-1 interactions in MDMi from individuals homozygous for the risk variant, rs3865444^CC^, compared to MDMi from individuals homozygous for the protective variant, rs3865444^AA^ (Figure 1).

### 3.2. Genotype-Specific CD33-SHP-1 Interactions in Post-Mortem Human Brain Tissue

**Figure 2:**
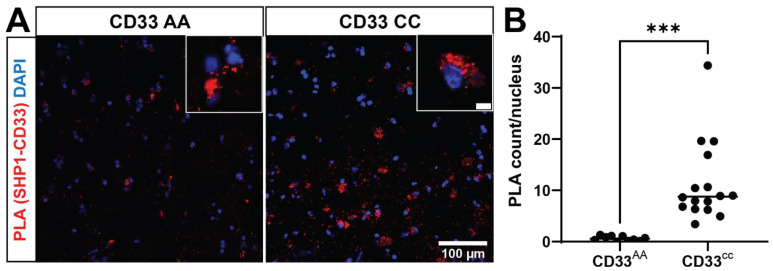
CD33 and SHP-1 interact in situ in a CD33 genotype-dependent manner. (**A**) A representative confocal image of post-mortem human prefrontal cortex stained for proximity ligation assays (PLA). The proximity ligation puncta (red dots, zoomed in inset) represent the protein–protein interaction between CD33 and SHP-1. DAPI (blue) is used to counterstain the nucleus. (**B**) Quantification of the PLA dots show a significant increase in the PLA counts in the CD33 CC group (*n* = 16 tissue sections, 11.36 ± 1.956) when compared to the CD33 AA groups (*n* = 10 tissue sections, 0.6185 ± 0.1482). Data represented as the mean ± SEM. *** *p* < 0.001, unpaired *t*-test. Scale bar: 100 μm; 10 μm inset.

We next tested whether SHP-1 colocalizes with CD33-positive cells in human prefrontal cortex. By utilizing an immunofluorescence technique in frozen human brain coupled with confocal microscopy, we found colocalization of SHP-1 and CD33, suggesting these proteins have the potential to interact (Supplemental Figure S2). To confirm whether the CD33 and SHP-1 positive cells also express IBA1, we performed triple staining with CD33, SHP-1, and IBA1 in formalin fixed paraffin embedded tissue. We found that many of the CD33-positive cells also express IBA1, and that there are triple-positive cells (Supplemental Figure S3). To determine whether CD33 and SHP-1 interact, we performed in situ PLA in frozen post-mortem human brain tissues (prefrontal cortex) from five donors (Supplemental Table S1). We found 18.4 times more PLA signal in the rs3865444^CC^ group compared to the rs3865444^AA^ group (Figure 2), mirroring our genotype-specific findings observed in MDMi. This finding suggests that not only are CD33 and SHP-1 within the same biological complex of <40 nm and interacting to signal downstream functions, but their interaction is dictated by genotype in situ. The increased interaction in situ, even without pervanadate treatment, may be due to a modified activation state of SHP-1 in the brain of individuals with the rs3865444^CC^ genotype, suggesting the importance of molecular changes throughout the AD brain on microglial signaling.

### 3.3. CD33 and PTPN6 Gene-Expression Interaction Impacts the Risk for Clinical and Pathological Features of AD

To examine the implications of the interaction between CD33 and SHP-1 on disease, we examined gene–gene, SNP–gene, and mediation interactions in a transcriptomics dataset of frontal cortex from the ROSMAP cohort, a longitudinal cohort of aging [26]. To understand the biological significance of this interaction in the context of Alzheimer’s disease, we leveraged existing ROSMAP dorsolateral prefrontal cortex (DLPFC) transcriptomic and genetic datasets and investigated whether the interactions between *CD33* and *PTPN6* gene expression influence AD clinical and pathological traits. After adjustment for multiple testing correction for testing eight traits, *CD33-PTPN6* interaction was significantly associated with amyloid burden, tangles, pathological diagnosis of AD, and global burden of AD pathology. Their interaction was also nominally significant with an antemortem diagnosis of clinical AD (Table 1). This is supportive of the idea that CD33 requires SHP-1 to signal in immune cells. We repeated the interaction analysis for *CD33* and *PTPN11*, which codes for SHP-2, another protein tyrosine phosphatase that has been shown to bind to the ITIM motif of CD33 [16]. Interestingly, the *CD33* and *PTPN11* interaction was not associated with any of the eight traits (Supplemental Table S2).

To examine SNP–gene interactions, we performed trans-expression quantitative trait loci (eQTL) analysis. We tested the influence of six SNPs in the *PTPN6* locus on *CD33* expression and 57 SNPs in the *CD33* locus and found no trans-eQTLs of gene expression in either gene (Supplemental Tables S3 and S4). Additionally, *PTPN11* variants do not regulate the expression of *CD33* and vice versa (Supplemental Tables S5 and S6).

We then performed causal mediation analysis to test whether *PTPN6* gene expression mediates the association between *CD33* gene expression and AD-related traits, or vice versa. *PTPN6* gene expression did not mediate any associations between *CD33* and any traits (Supplemental Table S7). However, *CD33* gene expression was found to mediate the association between *PTPN6* gene expression and clinical AD dementia. These findings suggest that *PTPN6* gene expression affects *CD33* gene expression, and, in turn, the *CD33* gene expression affects AD dementia (*p* = 0.008), while *PTPN6* gene expression is not directly associated with AD dementia (Supplemental Table S8). To examine this further, we used mediation analysis to determine that *CD33* gene expression mediated the association of *PTPN6* gene expression on AD dementia (Table 2). The causal mediation analysis with *CD33* and *PTPN11* found no significant mediation effect of either gene with any AD traits (Supplemental Tables S9 and S10). This finding supports the idea that the mediation of *PTPN6* by *CD33* is specific to *PTPN6* in terms of CD33 signaling.

## 4. Discussion

In this study, we showed that genotype-dependent interactions between CD33 and SHP-1 in human microglia and microglia-like cells provide novel insights into the molecular mechanisms underlying Alzheimer’s disease (AD) susceptibility. The data demonstrate that the AD-associated *CD33* rs3865444^CC^ risk allele modulates the interaction between CD33 and SHP-1 in a phosphorylation-sensitive manner, potentially contributing to altered microglial function in AD. Additionally, we found a significant interaction between *CD33* and *PTPN6* (the gene encoding SHP-1) gene expression that influences various AD-related traits, suggesting a complex molecular interplay impacting AD pathology.

**Figure 3:**
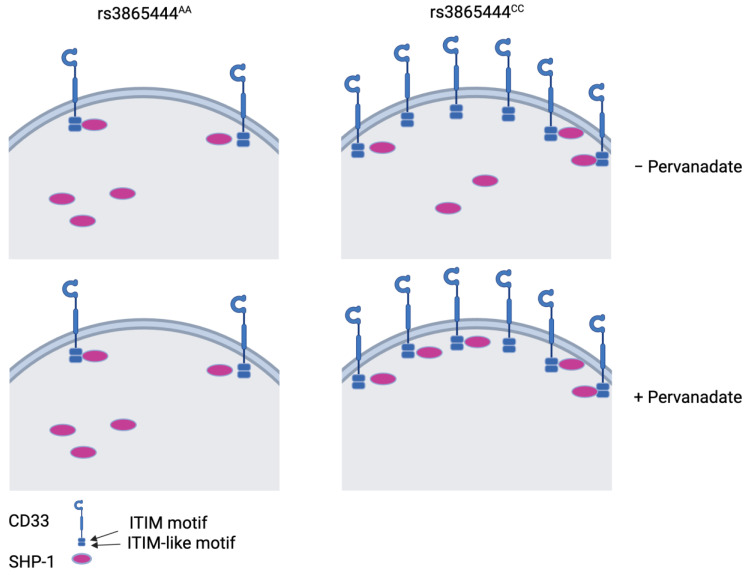
We hypothesize that microglia with the protective AA genotype have a limited number of CD33 ITIM motifs available for SHP-1 binding, while microglia from individuals with the risk CC genotype have a higher availability of ITIM motifs allowing for more SHP-1 recruitment during activation, as we observed with pervanadate treatment. This figure was made in BioRender.

Recent literature supports the critical role of innate immune cells in AD susceptibility, with *CD33* as a prominent genetic risk factor [14,35,36,37,38,39,40,41]. The *CD33* risk allele rs3865444^C^ increases full-length CD33 protein expression, leading to impaired microglial internalization of amyloid-β and ultimately amyloid plaque accumulation [14,15]. Our findings show that the CD33-SHP-1 interaction is phosphorylation sensitive and genotype influenced, suggesting that genotype-specific therapeutic strategies may be a relevant approach. Because the risk variant increases the expression of CD33 on the surface of immune cells (but not SHP-1 levels; Supplemental Figure S1) [14,19], the inability of pervanadate to increase CD33-SHP-1 binding in MDMi from individuals with the rs3865444^AA^ genotype suggests that, when CD33 is limited, there is high baseline occupancy of the ITIM motif with SHP-1. However, in the case of rs3865444^CC^ MDMi, there is an excess of CD33, and stimulation, especially with a protein tyrosine phosphatase inhibitor, can increase the occupancy of the ITIM motif with SHP-1 (Figure 3). Higher CD33-SHP-1 interaction in the brain with the *CD33* risk allele may result from chronic activation of CD33, maintaining SHP-1 in an active state and facilitating its downstream signaling, ultimately contributing to AD pathology.

Additionally, utilizing a large transcriptomic dataset, we were able to demonstrate that there are multiple pathological outcomes influenced by the gene–gene interaction of *CD33* and *PTPN6*. Specifically, we find that the interaction of *CD33* and *PTPN6* modulates the association with amyloid burden, tangles, pathological diagnosis of AD, and global burden of AD pathology. This demonstrates that CD33 and SHP-1/*PTPN6* are critical partners of a shared molecular pathway. Despite this strong association with pathology, the gene–gene interaction between *CD33* and *PTPN6* did not influence cognitive decline, TDP43, hippocampal sclerosis, and clinical diagnosis of AD, supporting the complexity of AD and microglial genes, as well as the concept that AD pathology is more than the buildup of amyloid plaques and tau tangles. Remarkably, when we performed the same analysis with *PTPN11* and *CD33,* we found no association with any of the traits. Since CD33 has been shown to be able to bind both SHP-1 and SHP-2, this suggests that there are in vivo binding affinity differences or that only the SHP-1 binding is linked to AD pathology.

Another interesting contrast between our protein and gene expression findings in regard to CD33-SHP-1 interaction is the role of genotype. Genetic variation at the *CD33* locus does not influence *PTPN6* gene expression, and genetic variation at the *PTPN6* locus does not influence *CD33* expression. However, the AD-associated genetic variation at the *CD33* locus does influence the interaction of CD33 and SHP-1 at the protein level.

Our study provides important insights, although there are a few limitations to consider. The sample size for the in situ analysis of post-mortem brain tissues was limited to five donors, so expanding the cohort in future studies would help validate our findings and enhance their generalizability. Additionally, while CD33 and IBA1 (the most commonly used immunohistochemical marker for microglia) often are found in the same cell, we did identify cells with microglial morphology expressing CD33 and not IBA1 and vice versa (Supplemental Figure S3). This is consistent with prior publications and suggests there is still much to explore about CD33′s expression in microglia, including the possibility of new microglial subpopulations or distinct activation states influenced by the local brain environment or disease context [38,42]. In our flow cytometry analysis of MDMi, we also see heterogeneity of CD33 protein expression with the majority having high expression and a smaller population having low CD33 expression, but consistent SHP-1 expression (Supplemental Figure S1).

Our study primarily focused on the phosphorylation state of SHP-1 and its interaction with CD33. Other post-translational modifications of SHP-1 or CD33, which might also influence their interaction, were not explored. Comprehensive studies considering various protein modifications are needed. While we demonstrated an interaction between CD33 and SHP-1, the functional consequences of this interaction in the context of microglial activity and AD progression were not fully elucidated. Further studies are needed to link these molecular interactions to specific cellular and pathological outcomes.

Given the genotype-dependent nature of the CD33-SHP-1 protein interaction, developing targeted therapies that modulate this interaction may be beneficial. Small molecule inhibitors or biologics that disrupt CD33-SHP-1 signaling in a controlled manner might be a novel therapeutic avenue. Expanding the scope of genetic interactions to include other innate immune receptors and their signaling partners could provide a more comprehensive understanding of the immune dysregulation in AD. Integrating multi-omic data from large cohorts could further elucidate these complex networks. Longitudinal studies tracking the progression of AD in relation to CD33 and SHP-1 interaction dynamics could provide valuable insights into how these interactions evolve with disease progression and influence clinical outcomes.

In conclusion, our study underscores the critical role of understanding the interaction partners and signaling pathways in microglia of genetically associated proteins, contributing significantly to the genetic risk of Alzheimer’s disease. These findings provide valuable insights into the molecular mechanisms underlying AD and suggest potential genotype-specific therapeutic strategies targeting the CD33-SHP-1 pathway. Future research should focus on further elucidating these interactions and exploring therapeutic interventions that can effectively modulate this pathway to mitigate AD progression. Continued investigation in this area holds promise for developing more effective treatments for this devastating disease.

## Figures and Tables

**Table 1:** Test for the interaction between *CD33* gene expression (gx) and *PTPN6* gene expression on trait.

Trait	Variable	β	se	t	*p*
**Amyloid**	Intercept	1.742	0.039	44.546	1.45 × 10^−246^
	*CD33* gx	0.070	0.056	1.236	0.217
	*PTPN6* gx	0.022	0.073	0.297	0.766
	*CD33* gx:*PTPN6* gx	−0.210	0.063	−3.349	**8.39 × 10^−4^**
**Tangles**	Intercept	2.347	0.047	49.816	4.20 × 10^−282^
	*CD33* gx	0.077	0.068	1.137	0.256
	*PTPN6* gx	−0.034	0.088	−0.383	0.701
	*CD33* gx:*PTPN6* gx	−0.247	0.076	−3.276	**1.09 × 10^−3^**
**Pathologic AD**	Intercept	0.644	0.072	8.889	6.18 × 10^−19^
	*CD33* gx	0.099	0.104	0.958	0.338
	*PTPN6* gx	0.038	0.135	0.280	0.780
	*CD33* gx:*PTPN6* gx	−0.359	0.114	−3.136	**1.72 × 10^−3^**
**AD dementia**	Intercept	0.163	0.084	1.945	0.052
	*CD33* gx	0.294	0.125	2.358	0.018
	*PTPN6* gx	−0.202	0.156	−1.289	0.197
	*CD33* gx:*PTPN6* gx	−0.299	0.146	−2.054	0.040
**Cognitive decline**	Intercept	−0.016	0.003	−4.804	1.78 × 10^−6^
	*CD33* gx	−0.007	0.005	−1.499	0.134
	*PTPN6* gx	0.001	0.006	0.217	0.828
	*CD33* gx:*PTPN6* gx	0.003	0.006	0.612	0.541
**TDP-43**	Intercept	−0.711	0.077	−9.259	2.05 × 10^−20^
	*CD33* gx	0.190	0.113	1.679	0.093
	*PTPN6* gx	−0.081	0.145	−0.562	0.574
	*CD33* gx:*PTPN6* gx	−0.296	0.140	−2.113	0.035
**Hippocampal sclerosis**	Intercept	−2.260	0.120	−18.778	1.1 × 410^−78^
	*CD33* gx	0.314	0.183	1.712	0.087
	*PTPN6* gx	−0.244	0.229	−1.067	0.286
	*CD33* gx:*PTPN6* gx	−0.279	0.227	−1.230	0.219
**Global AD pathology burden**	Intercept	0.760	0.021	35.835	2.32 × 10^−186^
	*CD33* gx	0.019	0.031	0.637	0.525
	*PTPN6* gx	0.033	0.040	0.831	0.406
	*CD33* gx:*PTPN6* gx	−0.123	0.034	−3.613	**3.16 × 10^−4^**

**Table 2:** Causal Mediation Analysis where the exposure is *PTPN6* gene expression, the mediator is *CD33* gene expression, and the outcome is AD dementia.

	β	Lower 95% CI	Upper 95% CI	*p*-Value
**Average Causal Mediation Effect (ACME)**	0.055	0.012	0.100	0.008
**Average Direct Effect (ADE)**	−0.042	−0.119	0.040	0.278
**Total Effect**	0.014	−0.045	0.070	0.666
**Proportion Mediated (PM)**	4.092	−22.768	27.120	0.670

## Data Availability

ROSMAP resources can be requested at https://www.radc.rush.edu and www.synpase.org, accessed on 11 September 2024.

## Supplementary Materials

The following supporting information can be downloaded at: https://www.mdpi.com/article/10.3390/genes15091204/s1, Supplemental Methods [19,43,44]; Figure S1: Most MDMi cells express both CD33 and SHP-1; Figure S2: A representative image of frozen human brain (prefrontal cortex) immunostained with CD33 and SHP-1; Figure S3: Representative images of the mid-frontal cortex immunostained with CD33, SHP-1, and IBA1; Table S1: Diagnosis and rs3865444 genotype of samples used in Figure 2; Table S2: Test for the interaction between *CD33* gene expression and *PTPN11* gene expression on trait; Table S3: Test for the effect of *PTPN6* SNPs on *CD33* gene expression; Table S4: Test for the effect of *CD33* SNPs on *PTPN6* gene expression; Table S5: Test for the effect of *PTPN11* SNPs on *CD33* gene expression; Table S6: Test for the effect of *CD33* SNPs on *PTPN11* gene expression; Table S7: Causal Mediation Analysis. Test whether *PTPN6* gene expression mediates the association between *CD33* gene expression and a trait; Table S8: Causal Mediation Analysis. Test whether *CD33* gene expression mediates the association between *PTPN6* gene expression and trait; Table S9: Causal Mediation Analysis. Test whether *PTPN11* gene expression mediates the association between *CD33* gene expression and trait; Table S10: Causal Mediation Analysis. Test whether *CD33* gene expression mediates the association between *PTPN11* gene expression and trait.
