# Supplementary material for: CD33 and SHP-1/PTPN6 Interaction in Alzheimer’s Disease
Source: Genes (Basel). 2024 Sep 13;15(9):1204. doi: 10.3390/genes15091204 (PMC11431297; doi:10.3390/genes15091204)
Supplement: Supplementary file 1 [file genes-15-01204-s001.zip › genes-3108681-supplementary.pdf]

Supplementary Methods and Figures:

Protein Quantitation in MDMi

Flow cytometry was used to validate protein expression of both CD33 and SHP-1 in MDMi (**Supplemental Figure S1**). CD14+ monocytes were isolated from PBMCs of healthy individuals with either rs3865444^{CC} or rs3865444^{AA} genotypes. Cells were plated in a round-bottom, polypropylene 96-well plate at a density of 200,000 cells in 200 µL of differentiation media. After 10 days of differentiation into MDMi, cells were incubated with TruStain FcX Human (Biolegend 422302) on ice for 10 min. Cells were then washed with staining buffer (1% FBS in PBS), pelleted at 2000 rpm for 10 min at 4°C, then incubated on ice with CD33-APC conjugated antibody (Miltenyi 130-113-345) for 20 min. After incubation, cells were washed twice with staining buffer and fixed with Intracellular Fixation Buffer (Invitrogen 88-8824-00) for 30 min on ice. Cells were then washed with Intracellular Permeabilization Buffer (Invitrogen 88-8824-00) and incubated with SHP1-PE conjugated antibody (Abcam ab209913) (prepared in permeabilization buffer) for 30 min on ice. Finally, cells were washed once with permeabilization buffer, once with staining buffer, and then stored in 100 µL of staining buffer at 4°C protected from light. Flow cytometry was performed the next day on a NovoCyte Penteon Flow Cytometer.

Preparation and immunohistochemistry of human brain tissue

Human post-mortem brain specimens used for immunohistochemistry (IHC) and proximity ligation assay (PLA) were obtained from the New York Brain Bank (NYBB) from the Columbia University Irving Medical Center ADRC and were accrued and processed per its published protocol[1]. Immunohistochemistry was performed on frozen sections of human prefrontal cortex. Sections were fixed in 100% ethanol for 15 minutes at -20°C after thawing them for 10 minutes. Slides were washed three times with 1X PBS. The sections were then blocked with 3% BSA in 1X PBS

containing 0.1% Triton-X for 1 hour at room temperature. Primary antibody in 1% BSA in PBS was applied overnight at 4°C. Primary antibodies used were mouse anti-SHP-1 (Thermo Fisher, Cat# MA5-11669); rabbit anti-CD33 (Sigma, Cat# HPA035832). After washing the slides three times with PBS, a secondary antibody in PBS was added for 1 hour at room temperature. The secondary antibodies used were goat anti-mouse IgG (H+L) conjugated to Alexa Fluor Plus 488 (Thermo Fisher; Cat # A32723; 1:300) and goat anti-rabbit IgG (H+L) conjugated to Alexa Fluor Plus 555 (Thermo Fisher; Cat# A32732; 1:300). Slides were washed three times and subsequently incubated with 0.3% Sudan Black in 70% ethanol for 10 minutes. After thoroughly washing with PBS, the slides were mounted with ProLong Gold with DAPI (Thermo Fisher, Cat # P36931) (**Supplemental Figure S2**).

Immunohistochemistry, Imaging and analysis of FFPE tissue

In this study, formalin-fixed paraffin-embedded (FFPE) mid-frontal cortical tissue sections were used which were collected from the ROSMAP study. The IHC methods were adopted from our previously published protocol[2]. To deparaffinize the sections, they were first treated with CitriSolv (Decon Labs #1601H) for three minutes, followed by two washes in 100% ethanol for one minute each and a subsequent wash in 70% ethanol for one minute. The sections were then rinsed three times with PBS. Antigen retrieval was performed by microwaving the sections in citrate buffer for 20 minutes, followed by three PBS washes. The sections were then blocked using a solution of 5% donkey serum and 0.1% Triton X-100 in PBS. For immunostaining, the sections were incubated overnight at 4°C with the following primary antibodies for multiplexing: IBA1 (Wako, Cat # 011-2799, 1:250), CD33 (Millipore Sigma, Cat# HPA035832, 1:200), SHP-1 (Thermo Fisher, Cat# LF-MA0212, 1:100). After the immunostaining, Trueblack (Biotium, Cat# 23007) was used to reduce the autofluorescence. Sections were mounted in Antifade Mountant (Invitrogen, cat # P36984) under coverslips (No.1.5, VWR) for subsequent microscopy. Confocal images were

acquired using Zeiss LSM 900 confocal laser scanning microscope (The Microscopy core of the Columbia Center for Translational Immunology, Columbia University Irving Medical Center) using Plan-Apochromat 20x/0.8 M27 objective with a 512 X 512 pixel resolution with z-stack of step size 1µm. Images were analyzed using Image J software, where average intensity of the projected z-stack were used (**Supplemental Figure S3**).

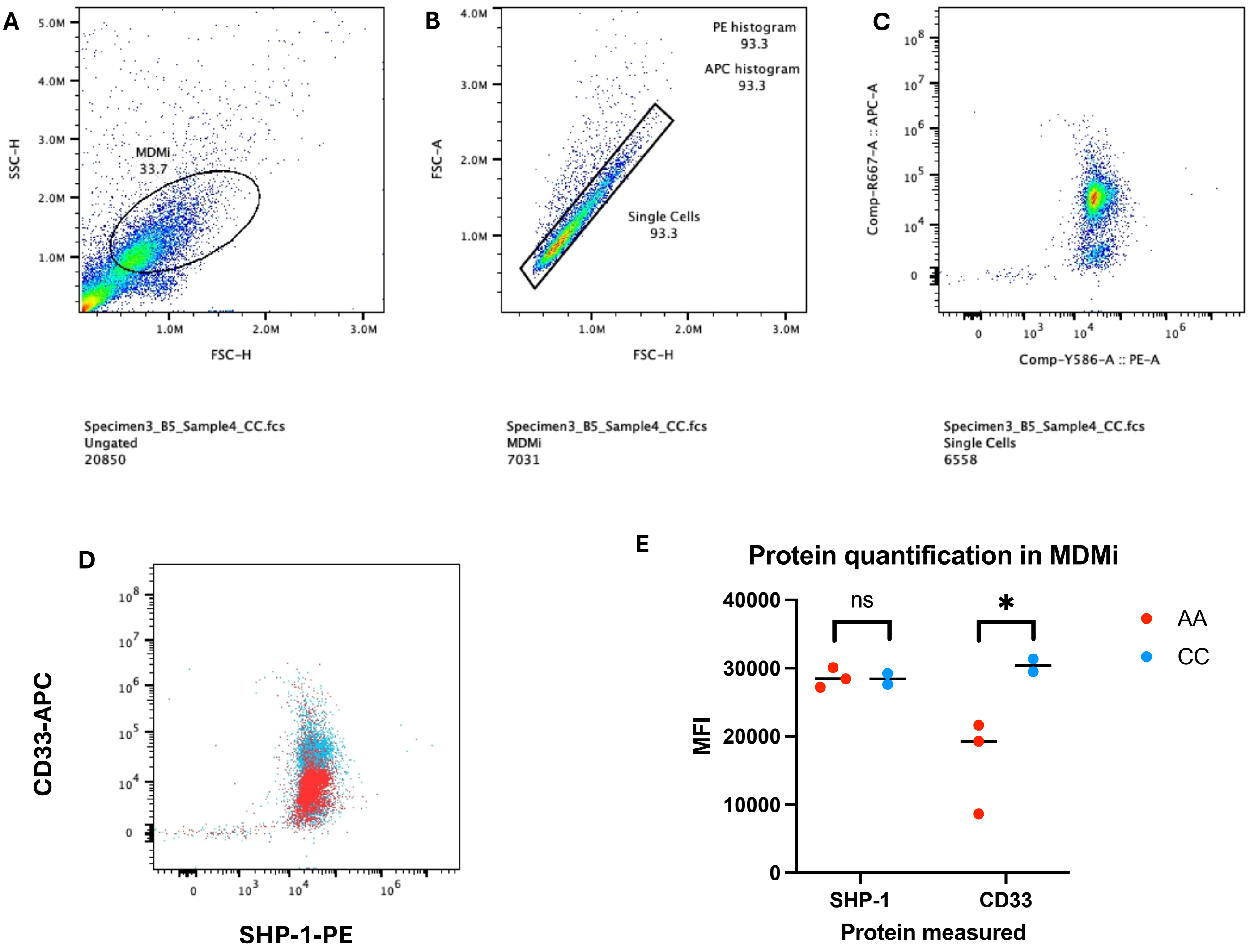

Supplemental Figure S1: Most MDMi cells express both CD33 and SHP-1. (A-C) Gating strategy for MDMi. (D) Overlay of MDMi from a rs3865444^{AA} person (red) and MDMi from a rs3865444^{CC} individual (blue). SHP-1-PE and CD33-APC. (E) MDMi have no difference in SHP-1 protein expression based on CD33 genotype, but as previously reported[3], CD33 expression is higher in individuals with the rs3865444^{CC} genotype. Each dot represents an individual. *$p<0.05$.

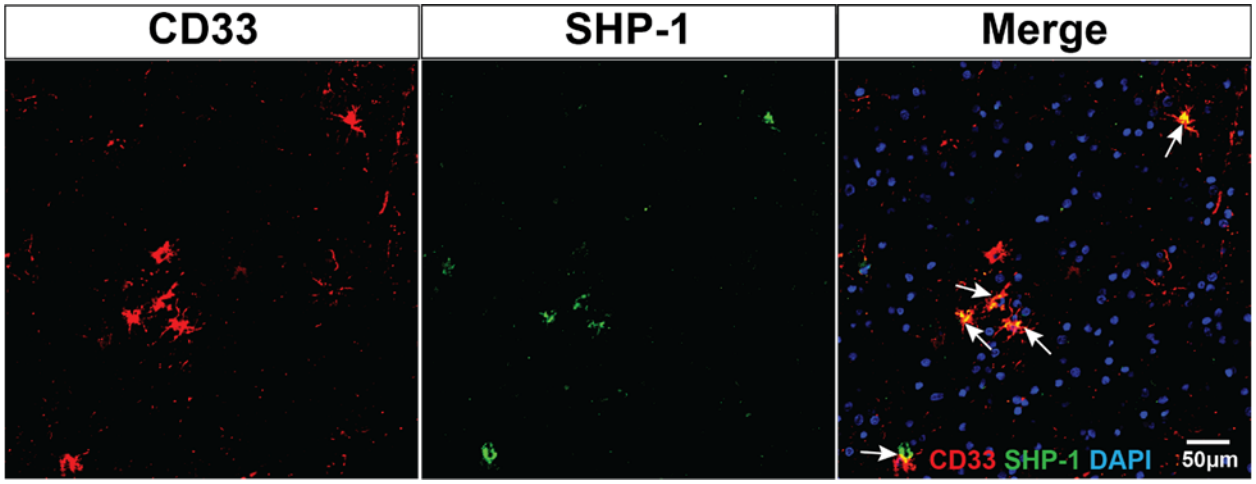

Supplementary Figure S2: A representative image of frozen human brain (prefrontal cortex) immunostained with CD33 and SHP-1. Red color indicates CD33 and green indicates SHP-1 immunosignals. The arrows show SHP-1 and CD33 overlapping pixels. DAPI (blue) is used to counterstain the nucleus. Scale bar: 50 μm.

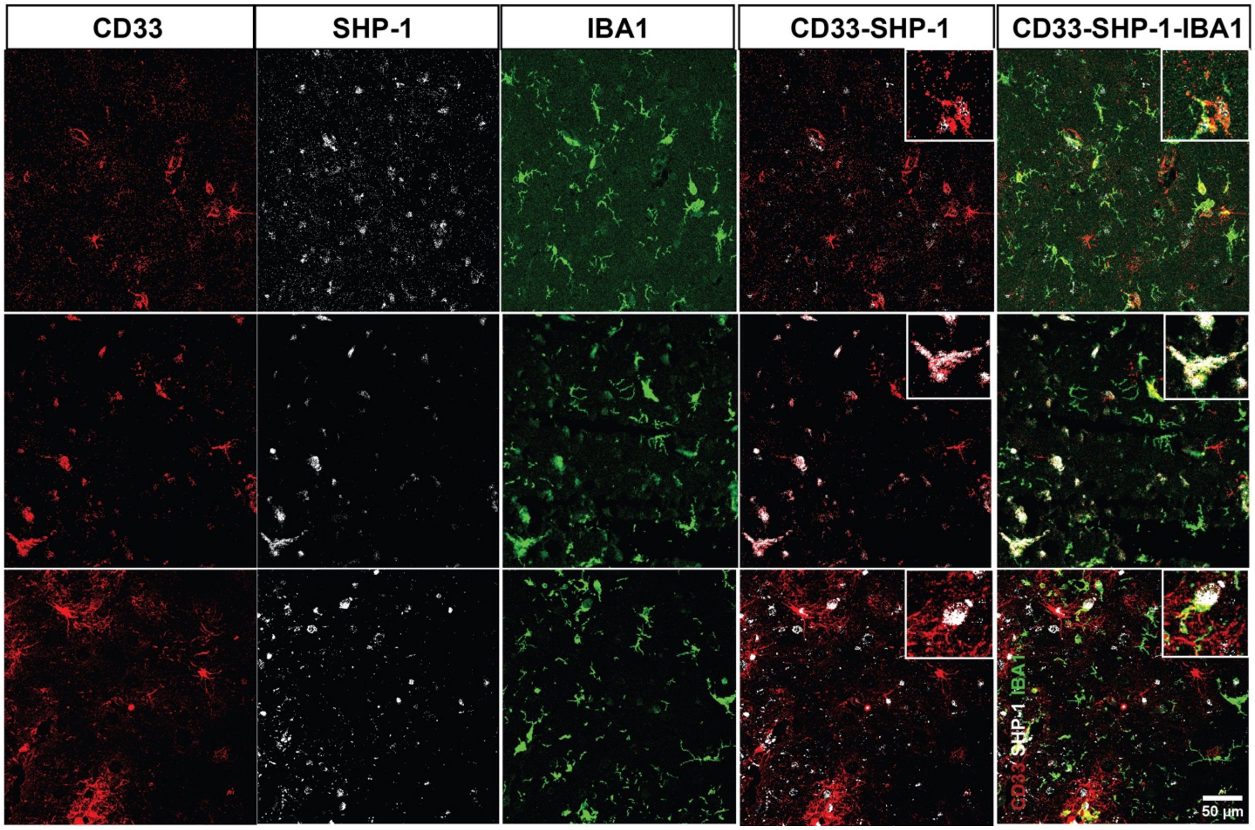

Supplementary Figure S3: Representative images of the mid-frontal cortex immunostained with CD33, SHP-1, and IBA1. CD33 is indicated in red, SHP-1 in white, and IBA1 in green. The inset shows a zoomed-in view of a cell representing either CD33-SHP-1 or CD33-SHP-1-IBA1. Scale bar: 50 µm.

Supp. Table S1

ID	Diagnosis	rs3865444 genotype
1	DLBD	AA
2	AD, LBD	AA
3	AD	CC
4	AD	CC
5	AD	CC

DLBD = Diffuse Lewy Body Disease
AD = Alzheimer's disease
LBD = Lewy body disease

Supp. Table S2

trait ~ CD33 gx + PTPN11 gx + CD33 gx x PTPN11 gx

Test for the interaction between CD33 gx and PTPN11 gx on trait

**trait**	**variable**	**b**	**se**	**t**	**p**
amyloid	CD33gx:PTPN11gx	0.24355879	0.17123158	1.42239405	0.15520144
tangles	CD33gx:PTPN11gx	0.09063946	0.20698537	0.43790271	0.66154438
pathologic AD	CD33gx:PTPN11gx	0.50559672	0.31835566	1.58815058	0.11225229
AD dementia	CD33gx:PTPN11gx	0.27775343	0.3815654	0.72793138	0.46665559
cognitive decline	CD33gx:PTPN11gx	0.03041883	0.01580952	1.92408336	0.05462131
tdp43	CD33gx:PTPN11gx	-0.1218654	0.34517486	-0.3530542	0.72404781
hippocampal sclerosis	CD33gx:PTPN11gx	0.23848393	0.54520461	0.43742097	0.66180608
Global AD pathology burden	CD33gx:PTPN11gx	0.09126868	0.0932822	0.97841476	0.32808673

Supp. Table S3. Test for the effect of PTPN6 SNP on CD33
gx Model: CD33 gx ~ PTPN6 SNP + PC1 + PC2 + PC3

	All				stratified by male				stratified by female			
SNP	b	se	t	p	b	se	t	p	b	se	t	p
rs741090_A	0.050	0.041	1.223	0.222	0.027	0.075	0.362	0.718	0.055	0.049	1.139	0.255
rs4963514_A	0.040	0.074	0.534	0.593	0.203	0.140	1.448	0.149	-0.023	0.087	-0.263	0.792
rs56212533_A	-0.024	0.041	-0.582	0.560	-0.073	0.080	-0.921	0.358	0.002	0.048	0.051	0.960
rs2110072_T	0.069	0.073	0.938	0.349	0.245	0.139	1.764	0.079	0.001	0.086	0.015	0.988
rs2071079_G	0.027	0.038	0.701	0.483	0.077	0.069	1.115	0.266	0.004	0.045	0.086	0.932
rs73262897_A	0.036	0.064	0.559	0.577	0.042	0.116	0.364	0.716	0.024	0.076	0.313	0.754

Supp. Table S4. Test for the effect of CD33 SNP on PTPN6
gx Model: PTPN6 gx ~ CD33 SNP + PC1 + PC2 + PC3

	All				stratified by male				stratified by female			
SNP	b	se	t	p	b	se	t	p	b	se	t	p
rs117613660_A	-0.045	0.095	-0.478	0.633	0.183	0.170	1.077	0.282	-0.146	0.114	-1.278	0.202
rs3865444_A	0.009	0.029	0.309	0.757	-0.035	0.049	-0.707	0.480	0.031	0.037	0.848	0.397
rs2459141_C	-0.007	0.028	-0.249	0.804	0.090	0.047	1.921	0.056	-0.057	0.035	-1.632	0.103
rs117255828_T	-0.045	0.095	-0.478	0.633	0.183	0.170	1.077	0.282	-0.146	0.114	-1.278	0.202
rs12459419_T	0.008	0.029	0.273	0.785	-0.037	0.049	-0.762	0.447	0.031	0.037	0.840	0.401
rs2455069_G	-0.008	0.028	-0.296	0.768	0.088	0.047	1.868	0.063	-0.056	0.035	-1.621	0.105
rs7245846_A	0.016	0.029	0.561	0.575	-0.007	0.048	-0.150	0.881	0.026	0.036	0.736	0.462
rs74796228_A	-0.045	0.095	-0.478	0.633	0.183	0.170	1.077	0.282	-0.146	0.114	-1.278	0.202
rs33978622_C	0.017	0.029	0.600	0.549	-0.026	0.049	-0.539	0.590	0.039	0.036	1.069	0.285
rs73932887_A	-0.029	0.094	-0.310	0.757	0.183	0.170	1.077	0.282	-0.120	0.113	-1.064	0.288
rs34813869_G	0.012	0.029	0.425	0.671	-0.011	0.048	-0.230	0.818	0.022	0.036	0.629	0.530
rs73932888_C	-0.059	0.094	-0.625	0.532	0.183	0.170	1.077	0.282	-0.162	0.112	-1.438	0.151
rs1354106_G	0.011	0.029	0.367	0.713	-0.011	0.048	-0.230	0.818	0.020	0.036	0.557	0.578
rs35112940_A	0.010	0.033	0.289	0.773	0.008	0.055	0.142	0.887	0.008	0.042	0.196	0.845
rs10409348_A	-0.028	0.027	-1.015	0.310	-0.097	0.049	-2.005	**0.046**	0.004	0.033	0.106	0.916
rs1399839_C	-0.011	0.026	-0.428	0.669	0.063	0.045	1.422	0.156	-0.051	0.033	-1.562	0.119
rs273653_A	-0.013	0.026	-0.482	0.630	0.064	0.045	1.434	0.153	-0.054	0.033	-1.639	0.102
rs78347527_A	0.016	0.086	0.186	0.852	-0.049	0.171	-0.289	0.773	0.034	0.101	0.342	0.733
rs273652_C	0.022	0.027	0.820	0.412	-0.048	0.047	-1.029	0.304	0.057	0.033	1.720	0.086
rs1803254_C	-0.035	0.055	-0.631	0.528	-0.101	0.099	-1.021	0.308	-0.007	0.067	-0.108	0.914
rs8103085_T	-0.035	0.055	-0.631	0.528	-0.101	0.099	-1.021	0.308	-0.007	0.067	-0.108	0.914
rs1697531_G	0.024	0.027	0.875	0.382	-0.048	0.047	-1.029	0.304	0.059	0.033	1.786	0.075
rs1034521_G	-0.030	0.053	-0.555	0.579	-0.101	0.099	-1.021	0.308	-0.002	0.064	-0.028	0.978
rs12461354_A	-0.030	0.053	-0.555	0.579	-0.101	0.099	-1.021	0.308	-0.002	0.064	-0.028	0.978
rs76248157_T	-0.030	0.053	-0.555	0.579	-0.101	0.099	-1.021	0.308	-0.002	0.064	-0.028	0.978
rs73612300_C	-0.030	0.053	-0.555	0.579	-0.101	0.099	-1.021	0.308	-0.002	0.064	-0.028	0.978
rs273651_A	0.015	0.026	0.581	0.561	-0.064	0.045	-1.434	0.153	0.058	0.033	1.760	0.079
rs169275_T	0.024	0.027	0.877	0.381	-0.048	0.047	-1.029	0.304	0.059	0.033	1.785	0.075
rs273650_A	0.015	0.026	0.581	0.561	-0.064	0.045	-1.434	0.153	0.058	0.033	1.760	0.079
rs1566578_T	-0.030	0.053	-0.555	0.579	-0.101	0.099	-1.021	0.308	-0.002	0.064	-0.028	0.978
rs145323276_C	-0.015	0.058	-0.254	0.799	-0.073	0.112	-0.651	0.516	0.004	0.068	0.055	0.956
rs11084061_A	-0.030	0.053	-0.555	0.579	-0.101	0.099	-1.021	0.308	-0.002	0.064	-0.028	0.978
rs273649_C	0.024	0.027	0.877	0.381	-0.048	0.047	-1.029	0.304	0.059	0.033	1.785	0.075
rs11084062_C	-0.030	0.053	-0.555	0.579	-0.101	0.099	-1.021	0.308	-0.002	0.064	-0.028	0.978
rs12461282_C	-0.030	0.053	-0.555	0.579	-0.101	0.099	-1.021	0.308	-0.002	0.064	-0.028	0.978
rs273648_A	0.024	0.027	0.877	0.381	-0.048	0.047	-1.029	0.304	0.059	0.033	1.785	0.075
rs11084063_T	-0.028	0.053	-0.516	0.606	-0.101	0.099	-1.021	0.308	0.001	0.064	0.019	0.985
rs989504_T	-0.028	0.053	-0.516	0.606	-0.101	0.099	-1.021	0.308	0.001	0.064	0.019	0.985
rs273647_C	0.015	0.026	0.581	0.561	-0.064	0.045	-1.434	0.153	0.058	0.033	1.760	0.079
rs989502_A	-0.030	0.053	-0.555	0.579	-0.101	0.099	-1.021	0.308	-0.002	0.064	-0.028	0.978
rs989505_A	-0.030	0.053	-0.555	0.579	-0.101	0.099	-1.021	0.308	-0.002	0.064	-0.028	0.978
rs1319675_A	-0.030	0.053	-0.555	0.579	-0.101	0.099	-1.021	0.308	-0.002	0.064	-0.028	0.978
rs273646_T	0.024	0.027	0.877	0.381	-0.048	0.047	-1.029	0.304	0.059	0.033	1.785	0.075
rs273645_T	0.024	0.027	0.877	0.381	-0.048	0.047	-1.029	0.304	0.059	0.033	1.785	0.075
rs7253829_C	-0.030	0.053	-0.555	0.579	-0.101	0.099	-1.021	0.308	-0.002	0.064	-0.028	0.978
rs7253831_A	-0.030	0.053	-0.555	0.579	-0.101	0.099	-1.021	0.308	-0.002	0.064	-0.028	0.978
rs17801843_T	-0.030	0.053	-0.555	0.579	-0.101	0.099	-1.021	0.308	-0.002	0.064	-0.028	0.978
rs273644_A	0.024	0.027	0.872	0.384	-0.039	0.047	-0.824	0.410	0.054	0.033	1.644	0.101
rs273643_T	0.023	0.027	0.834	0.404	-0.047	0.048	-0.996	0.320	0.057	0.033	1.727	0.085
rs273642_C	-0.012	0.027	-0.456	0.649	0.063	0.045	1.405	0.161	-0.053	0.033	-1.598	0.111
rs273641_C	0.023	0.027	0.834	0.404	-0.047	0.048	-0.996	0.320	0.057	0.033	1.727	0.085
rs7256372_T	-0.036	0.053	-0.670	0.503	-0.101	0.099	-1.021	0.308	-0.011	0.064	-0.171	0.864
rs71358823_G	-0.079	0.067	-1.178	0.239	0.014	0.123	0.114	0.909	-0.124	0.081	-1.538	0.124
rs1501447_G	-0.036	0.053	-0.670	0.503	-0.101	0.099	-1.021	0.308	-0.011	0.064	-0.171	0.864
rs1501448_T	-0.036	0.053	-0.670	0.503	-0.101	0.099	-1.021	0.308	-0.011	0.064	-0.171	0.864
rs166653_G	0.024	0.027	0.876	0.381	-0.047	0.048	-0.996	0.320	0.059	0.033	1.778	0.076
rs273619_T	0.024	0.027	0.876	0.381	-0.047	0.048	-0.996	0.320	0.059	0.033	1.778	0.076

Supp. Table S5
CD33 gx ~ PTPN11 SNP + PC1 + PC2 + PC3
Test for the effect of PTPN11 SNP on CD33 gx

		All				stratified by male				stratified by female			
index	SNP	b	se	t	p	b	se	t	p	b	se	t	p
1	rs58805176_A	-0.0463	0.0669	-0.6916	0.48936	-0.1003	0.12165	-0.8249	0.41004	-0.0168	0.08005	-0.2098	0.83391
2	rs61941346_G	0.07071	0.07779	0.90899	0.36358	-0.038	0.14472	-0.2625	0.79307	0.10583	0.09219	1.14788	0.25143
3	rs73209627_T	0.20617	0.10868	1.89709	0.0581	0.24709	0.1941	1.27304	0.20391	0.17899	0.1312	1.36423	0.17295
4	rs117399332_C	0.06819	0.06434	1.05975	0.28951	0.00346	0.12473	0.02774	0.97789	0.10001	0.07501	1.33336	0.18287
5	rs73209628_G	0.00561	0.05628	0.09964	0.92065	-0.0734	0.10929	-0.6713	0.50248	0.03839	0.06533	0.58762	0.55698
6	rs75098304_G	0.06515	0.06391	1.01949	0.30822	-0.007	0.12392	-0.0568	0.95473	0.10067	0.07452	1.35096	0.17716
7	rs61941348_C	0.07214	0.07897	0.91352	0.36119	0.00545	0.14592	0.03737	0.97022	0.09251	0.09378	0.98651	0.32424
8	rs12582819_C	-0.0379	0.06707	-0.5652	0.57204	-0.1003	0.12165	-0.8249	0.41004	-0.0041	0.08036	-0.0513	0.95912
9	rs11066308_G	-0.0379	0.06707	-0.5652	0.57204	-0.1003	0.12165	-0.8249	0.41004	-0.0041	0.08036	-0.0513	0.95912
10	rs17822304_A	-0.0558	0.06766	-0.8252	0.40948	-0.1752	0.12681	-1.3815	0.16805	0.0055	0.07973	0.06896	0.94504
11	rs140358678_G	0.20267	0.10778	1.88029	0.06036	0.24709	0.1941	1.27304	0.20391	0.17407	0.12958	1.3434	0.1796
12	rs74237645_T	-0.0379	0.06707	-0.5652	0.57204	-0.1003	0.12165	-0.8249	0.41004	-0.0041	0.08036	-0.0513	0.95912
13	rs11612981_T	0.01625	0.05583	0.29111	0.77103	-0.0324	0.1063	-0.3048	0.76068	0.03839	0.06533	0.58762	0.55698
14	rs12423190_C	-0.0379	0.06707	-0.5652	0.57204	-0.1003	0.12165	-0.8249	0.41004	-0.0041	0.08036	-0.0513	0.95912
15	rs80279219_A	0.16682	0.0798	2.09053	0.03682	-0.0131	0.15474	-0.0848	0.93244	0.23617	0.09298	2.53989	0.01131
16	rs41279090_T	0.06174	0.06322	0.97663	0.32899	-0.007	0.12392	-0.0568	0.95473	0.0948	0.0734	1.29157	0.19695
17	rs112407300_T	0.06174	0.06322	0.97663	0.32899	-0.007	0.12392	-0.0568	0.95473	0.0948	0.0734	1.29157	0.19695
18	rs116999150_G	0.09546	0.08991	1.06178	0.28859	-0.0213	0.15806	-0.1346	0.89299	0.15009	0.10942	1.37173	0.17061
19	rs113499824_G	-0.0155	0.09338	-0.1665	0.8678	-0.1876	0.16805	-1.1161	0.2652	0.07817	0.11229	0.69616	0.48657
20	rs56768778_T	-0.0379	0.06707	-0.5652	0.57204	-0.1003	0.12165	-0.8249	0.41004	-0.0041	0.08036	-0.0513	0.95912
21	rs11066323_A	0.06174	0.06322	0.97663	0.32899	-0.007	0.12392	-0.0568	0.95473	0.0948	0.0734	1.29157	0.19695
22	rs41279092_A	0.07214	0.07897	0.91352	0.36119	0.00545	0.14592	0.03737	0.97022	0.09251	0.09378	0.98651	0.32424
23	rs112704007_C	-0.0647	0.12723	-0.5086	0.61111	0.09117	0.25243	0.36117	0.71821	-0.0989	0.14681	-0.674	0.50057
24	rs58116261_T	-0.031	0.06812	-0.4554	0.6489	-0.1034	0.12537	-0.8245	0.41026	0.00543	0.08099	0.06705	0.94656
25	rs61942266_G	0.07557	0.05727	1.31948	0.18731	0.0027	0.11358	0.02376	0.98106	0.10696	0.06608	1.61854	0.10602
26	rs76662479_T	-0.0332	0.09	-0.3689	0.71231	-0.1871	0.16257	-1.1511	0.25053	0.05118	0.1081	0.4734	0.63608
27	rs112732885_C	0.05087	0.06244	0.81471	0.41543	-0.0045	0.12097	-0.037	0.97049	0.07762	0.07279	1.06642	0.28662
28	rs114848118_A	0.20091	0.11071	1.81481	0.06985	0.23911	0.1992	1.20039	0.23086	0.17485	0.13317	1.31301	0.18963
29	rs117865528_T	0.05087	0.06244	0.81471	0.41543	-0.0045	0.12097	-0.037	0.97049	0.07762	0.07279	1.06642	0.28662
30	rs60902179_A	-0.028	0.06812	-0.4109	0.68121	-0.0956	0.12541	-0.7624	0.44638	0.00543	0.08099	0.06705	0.94656
31	rs12425405_C	-0.028	0.06812	-0.4109	0.68121	-0.0956	0.12541	-0.7624	0.44638	0.00543	0.08099	0.06705	0.94656
32	rs11066328_C	0.00806	0.06304	0.12783	0.89831	-0.0376	0.11915	-0.3154	0.75268	0.03157	0.07405	0.4263	0.67003
33	rs112865240_C	0.06508	0.05693	1.14311	0.25326	-0.0271	0.11149	-0.2435	0.80776	0.10327	0.06597	1.56556	0.11792
34	rs2158291_T	0.00806	0.06304	0.12783	0.89831	-0.0376	0.11915	-0.3154	0.75268	0.03157	0.07405	0.4263	0.67003
35	rs2107204_G	0.00771	0.06318	0.12198	0.90294	-0.0376	0.11915	-0.3154	0.75268	0.03103	0.07428	0.41777	0.67625
36	rs10850043_A	-0.0247	0.06794	-0.3629	0.71675	-0.0956	0.12541	-0.7624	0.44638	0.00988	0.08067	0.12252	0.90252
37	rs12424313_T	0.00771	0.06318	0.12198	0.90294	-0.0376	0.11915	-0.3154	0.75268	0.03103	0.07428	0.41777	0.67625
38	rs6489846_C	0.00771	0.06318	0.12198	0.90294	-0.0376	0.11915	-0.3154	0.75268	0.03103	0.07428	0.41777	0.67625
39	rs6489847_A	0.00273	0.06289	0.04336	0.96543	-0.0501	0.11751	-0.4261	0.67033	0.03103	0.07428	0.41777	0.67625
40	rs10744779_A	0.00771	0.06318	0.12198	0.90294	-0.0376	0.11915	-0.3154	0.75268	0.03103	0.07428	0.41777	0.67625
41	rs10850045_T	-0.0247	0.06794	-0.3629	0.71675	-0.0956	0.12541	-0.7624	0.44638	0.00988	0.08067	0.12252	0.90252
42	rs111354741_T	0.04301	0.06219	0.69166	0.48931	-0.0264	0.12007	-0.2203	0.82581	0.07525	0.07257	1.03687	0.30017
43	rs75953948_A	-0.0491	0.05061	-0.9694	0.33259	-0.1344	0.09339	-1.4397	0.15091	-0.0039	0.06008	-0.0642	0.94883
44	rs11066330_A	-0.0247	0.06794	-0.3629	0.71675	-0.0956	0.12541	-0.7624	0.44638	0.00988	0.08067	0.12252	0.90252
45	rs2891397_G	-0.0343	0.06794	-0.5052	0.6135	-0.0956	0.12645	-0.7563	0.45001	-0.0068	0.08035	-0.0846	0.93259
46	rs11615234_A	0.03505	0.04269	0.82105	0.41181	-0.0497	0.08415	-0.5904	0.55533	0.06993	0.04933	1.41759	0.15678
47	rs10774654_G	-0.0056	0.06289	-0.0883	0.92964	-0.0494	0.11833	-0.4175	0.67659	0.01673	0.07405	0.22593	0.82133
48	rs77074373_G	-0.031	0.09053	-0.3423	0.7322	-0.1908	0.16531	-1.1544	0.2492	0.05118	0.1081	0.4734	0.63608
49	rs4767879_G	-0.0007	0.06318	-0.0103	0.99177	-0.0367	0.11997	-0.3058	0.75995	0.01673	0.07405	0.22593	0.82133
50	rs4767880_G	-0.0007	0.06318	-0.0103	0.99177	-0.0367	0.11997	-0.3058	0.75995	0.01673	0.07405	0.22593	0.82133
51	rs10850047_T	-0.0343	0.06794	-0.5052	0.6135	-0.0956	0.12645	-0.7563	0.45001	-0.0068	0.08035	-0.0846	0.93259
52	rs2383999_A	-0.0343	0.06794	-0.5052	0.6135	-0.0956	0.12645	-0.7563	0.45001	-0.0068	0.08035	-0.0846	0.93259
53	rs147001808_T	-0.0026	0.05639	-0.0469	0.96257	-0.0551	0.10685	-0.5159	0.60626	0.02187	0.06612	0.3308	0.7409
54	rs2384000_G	-0.0007	0.06318	-0.0103	0.99177	-0.0367	0.11997	-0.3058	0.75995	0.01673	0.07405	0.22593	0.82133
55	rs12231509_C	-0.0056	0.06289	-0.0883	0.92964	-0.0494	0.11833	-0.4175	0.67659	0.01673	0.07405	0.22593	0.82133
56	rs7136364_C	0.03336	0.04289	0.77768	0.43694	-0.0611	0.08435	-0.7248	0.46911	0.07261	0.04963	1.46314	0.1439
57	rs10850048_T	-0.0343	0.06794	-0.5052	0.6135	-0.0956	0.12645	-0.7563	0.45001	-0.0068	0.08035	-0.0846	0.93259
58	rs10850049_G	-0.0007	0.06318	-0.0103	0.99177	-0.0367	0.11997	-0.3058	0.75995	0.01673	0.07405	0.22593	0.82133
59	rs10850050_C	-0.0007	0.06318	-0.0103	0.99177	-0.0367	0.11997	-0.3058	0.75995	0.01673	0.07405	0.22593	0.82133
60	rs10850051_C	-0.0007	0.06318	-0.0103	0.99177	-0.0367	0.11997	-0.3058	0.75995	0.01673	0.07405	0.22593	0.82133
61	rs79582004_A	-0.0343	0.06794	-0.5052	0.6135	-0.0956	0.12645	-0.7563	0.45001	-0.0068	0.08035	-0.0846	0.93259

62	rs10850052_A	-0.0007	0.06318	-0.0103	0.99177	-0.0367	0.11997	-0.3058	0.75995	0.01673	0.07405	0.22593	0.82133
63	rs61942267_C	0.06892	0.05701	1.20878	0.22703	-0.0134	0.11207	-0.1196	0.90487	0.10327	0.06597	1.56556	0.11792
64	rs113589536_G	0.16074	0.07776	2.06724	0.03897	0.0092	0.15266	0.06029	0.95196	0.21704	0.09017	2.40693	0.01636
65	rs73209645_A	0.19635	0.10964	1.79094	0.0736	0.20734	0.19388	1.06943	0.28566	0.17485	0.13317	1.31301	0.18963
66	rs73209646_C	-0.0735	0.07028	-1.0454	0.29608	-0.2009	0.12652	-1.5881	0.11324	-0.0025	0.08443	-0.0293	0.97663
67	rs148745582_G	0.1423	0.13954	1.01978	0.30808	0.22274	0.26396	0.84382	0.39939	0.12105	0.16381	0.73899	0.46017
68	rs75513498_T	-0.0318	0.10861	-0.2924	0.77002	-0.3932	0.19389	-2.0279	0.04338	0.14467	0.13057	1.10796	0.26828
69	rs73209650_T	0.00429	0.0543	0.07896	0.93708	0.0044	0.1018	0.04325	0.96553	0.0036	0.06396	0.05626	0.95515
70	rs11066344_T	-0.0152	0.06777	-0.2249	0.82211	-0.0885	0.12571	-0.7044	0.4817	0.01814	0.08023	0.22616	0.82114
71	rs7302596_T	0.00429	0.0543	0.07896	0.93708	0.0044	0.1018	0.04325	0.96553	0.0036	0.06396	0.05626	0.95515
72	rs11613451_A	0.21728	0.10968	1.98104	0.04786	0.21051	0.20441	1.02987	0.30383	0.20792	0.12982	1.60159	0.10972
73	rs7975817_C	-0.0073	0.04519	-0.1606	0.87246	-0.0369	0.08441	-0.4367	0.66263	0.0054	0.05331	0.10137	0.91929
74	rs117972551_T	0.14948	0.1395	1.07156	0.28418	0.26563	0.27762	0.95682	0.33937	0.11954	0.16044	0.74506	0.4565
75	rs11615047_C	0.01949	0.0537	0.36294	0.71673	0.04257	0.10047	0.42369	0.67207	0.00982	0.06331	0.15514	0.87676
76	rs4767908_C	-0.0322	0.06897	-0.4665	0.64099	-0.1561	0.12869	-1.2132	0.22592	0.02109	0.0814	0.25908	0.79565
77	rs78159862_T	0.14948	0.1395	1.07156	0.28418	0.26563	0.27762	0.95682	0.33937	0.11954	0.16044	0.74506	0.4565
78	rs57675606_T	0.00429	0.0543	0.07896	0.93708	0.0044	0.1018	0.04325	0.96553	0.0036	0.06396	0.05626	0.95515
79	rs6489849_T	-0.0034	0.04502	-0.0762	0.93928	-0.0369	0.08441	-0.4367	0.66263	0.01078	0.05302	0.20341	0.83888
80	rs141944669_T	-0.0635	0.06467	-0.9814	0.32663	-0.0366	0.11418	-0.3206	0.74875	-0.0745	0.07855	-0.948	0.34346
81	rs7974244_A	-0.0034	0.04502	-0.0762	0.93928	-0.0369	0.08441	-0.4367	0.66263	0.01078	0.05302	0.20341	0.83888
82	rs117149835_C	-0.0225	0.15681	-0.1437	0.8858	0.53342	0.29504	1.80797	0.07153	-0.2743	0.18438	-1.4876	0.13731
83	rs12298862_C	-0.0533	0.06483	-0.8216	0.41149	-0.0366	0.11418	-0.3206	0.74875	-0.0585	0.07886	-0.7416	0.45861
84	rs113907453_T	0.07237	0.13383	0.54076	0.5888	0.26563	0.27762	0.95682	0.33937	0.02055	0.1517	0.13546	0.89229
85	rs11066353_A	0.02958	0.04929	0.6002	0.54851	0.02475	0.09473	0.26131	0.79402	0.03651	0.05754	0.63442	0.52602
86	rs117398984_T	0.14948	0.1395	1.07156	0.28418	0.26563	0.27762	0.95682	0.33937	0.11954	0.16044	0.74506	0.4565
87	rs118162036_T	0.14948	0.1395	1.07156	0.28418	0.26563	0.27762	0.95682	0.33937	0.11954	0.16044	0.74506	0.4565
88	rs73193163_A	0.18184	0.10513	1.72959	0.08401	0.08276	0.19427	0.42599	0.6704	0.21885	0.12482	1.75334	0.08
89	rs117748100_G	0.08624	0.11643	0.74073	0.45903	0.25005	0.21708	1.15187	0.25022	0.0168	0.13744	0.12225	0.90274
90	rs117067694_T	0.08624	0.11643	0.74073	0.45903	0.25005	0.21708	1.15187	0.25022	0.0168	0.13744	0.12225	0.90274
91	rs66475155_G	0.01044	0.05429	0.19222	0.84761	-0.0063	0.10134	-0.0623	0.95033	0.01794	0.06409	0.28	0.77956
92	rs7975499_C	0.01044	0.05429	0.19222	0.84761	-0.0063	0.10134	-0.0623	0.95033	0.01794	0.06409	0.28	0.77956
93	rs74672796_A	0.01485	0.0597	0.24867	0.80366	-0.1207	0.11881	-1.0155	0.31061	0.06493	0.06867	0.94557	0.34471
94	rs10850061_T	-0.0129	0.06635	-0.1938	0.84638	-0.0857	0.12437	-0.6891	0.49123	0.02051	0.07816	0.26238	0.79311
95	rs57485365_C	0.01044	0.05429	0.19222	0.84761	-0.0063	0.10134	-0.0623	0.95033	0.01794	0.06409	0.28	0.77956
96	rs7305492_T	0.01044	0.05429	0.19222	0.84761	-0.0063	0.10134	-0.0623	0.95033	0.01794	0.06409	0.28	0.77956
97	rs189997580_G	0.0084	0.10148	0.08274	0.93407	0.17141	0.18823	0.91061	0.36317	-0.0588	0.12015	-0.4894	0.62469
98	rs4766971_A	-0.0129	0.06635	-0.1938	0.84638	-0.0857	0.12437	-0.6891	0.49123	0.02051	0.07816	0.26238	0.79311
99	rs112296760_G	0.01978	0.05815	0.34013	0.73383	-0.0656	0.11084	-0.5918	0.55438	0.05677	0.06795	0.83551	0.40373
100	rs67116570_C	0.01357	0.05671	0.23934	0.81089	-0.0637	0.1078	-0.5908	0.55505	0.04664	0.06635	0.70286	0.48239
101	rs1859245_G	0.01357	0.05671	0.23934	0.81089	-0.0637	0.1078	-0.5908	0.55505	0.04664	0.06635	0.70286	0.48239
102	rs16942117_G	0.01297	0.05678	0.22843	0.81936	-0.0637	0.1078	-0.5908	0.55505	0.04653	0.0665	0.69968	0.48437
103	rs79422064_T	-0.0162	0.10076	-0.1608	0.8723	0.17141	0.18823	0.91061	0.36317	-0.094	0.1189	-0.7907	0.4294
104	rs11612310_G	0.01632	0.0566	0.28833	0.77315	-0.0637	0.1078	-0.5908	0.55505	0.05099	0.06617	0.77059	0.44122
105	rs11610244_A	0.01632	0.0566	0.28833	0.77315	-0.0637	0.1078	-0.5908	0.55505	0.05099	0.06617	0.77059	0.44122
106	rs11831995_T	0.15143	0.10595	1.42928	0.15324	0.0932	0.20462	0.45547	0.64907	0.16692	0.12338	1.35291	0.17654
107	rs73193171_A	-0.0562	0.07147	-0.7862	0.43195	-0.1337	0.12671	-1.0548	0.2923	-0.0082	0.08671	-0.0948	0.92452
108	rs11832547_T	0.18653	0.10506	1.77535	0.07614	0.0932	0.20462	0.45547	0.64907	0.21559	0.12192	1.76835	0.07746
109	rs112293504_T	-0.0683	0.07026	-0.9726	0.33098	-0.1337	0.12671	-1.0548	0.2923	-0.0297	0.0845	-0.3519	0.72501
110	rs112505310_G	-0.0487	0.05889	-0.8271	0.40838	-0.0484	0.10485	-0.4612	0.64497	-0.045	0.07131	-0.6308	0.52839
111	rs67436553_A	0.1325	0.10598	1.25028	0.21149	0.08876	0.19924	0.44549	0.65626	0.14457	0.12488	1.15765	0.24742
112	rs233724_A	0.03695	0.03588	1.02988	0.30331	0.02645	0.0679	0.38959	0.69709	0.03997	0.0422	0.94708	0.34394
113	rs117367092_C	0.09006	0.10569	0.8521	0.39436	0.26846	0.17765	1.51113	0.13172	-0.025	0.13232	-0.1887	0.85039
114	rs61942273_A	0.04746	0.03551	1.33642	0.18171	0.07855	0.06217	1.2634	0.20734	0.02986	0.04335	0.68887	0.49115
115	rs233723_A	-0.0142	0.04507	-0.3159	0.75212	-0.1135	0.08353	-1.3585	0.17524	0.02798	0.05333	0.5247	0.59996
116	rs233722_G	-0.0458	0.03564	-1.285	0.19908	-0.0368	0.06497	-0.5664	0.57153	-0.0453	0.04274	-1.061	0.28908
117	rs233721_T	-0.055	0.03657	-1.5027	0.13324	-0.034	0.06824	-0.4984	0.61856	-0.0604	0.04344	-1.3903	0.16489
118	rs56098743_T	0.0672	0.03549	1.89365	0.05856	0.09639	0.06181	1.55946	0.11985	0.04869	0.04347	1.11994	0.26314
119	rs233720_C	-0.0153	0.04505	-0.3386	0.73497	-0.1135	0.08353	-1.3585	0.17524	0.02633	0.0533	0.49403	0.62145
120	rs233719_T	-0.0156	0.04681	-0.3338	0.7386	-0.1281	0.08669	-1.4774	0.14052	0.03354	0.05539	0.60551	0.54505
121	rs61942275_T	0.06117	0.03548	1.72405	0.08501	0.09639	0.06181	1.55946	0.11985	0.03963	0.04345	0.91212	0.36204
122	rs17824032_T	0.05994	0.03543	1.69174	0.09101	0.09267	0.0616	1.50441	0.13344	0.03963	0.04345	0.91212	0.36204
123	rs17824050_G	0.05994	0.03543	1.69174	0.09101	0.09267	0.0616	1.50441	0.13344	0.03963	0.04345	0.91212	0.36204
124	rs233718_G	-0.0165	0.04488	-0.3678	0.71313	-0.1151	0.08253	-1.3942	0.16422	0.02633	0.0533	0.49403	0.62145
125	rs233716_C	-0.0458	0.03583	-1.2778	0.20163	-0.03	0.06486	-0.4621	0.64431	-0.0491	0.04311	-1.1396	0.25487
126	rs35730640_G	-0.0653	0.10317	-0.6327	0.5271	-0.0774	0.21153	-0.3659	0.71464	-0.0588	0.11749	-0.5004	0.61694
127	rs55847700_A	0.08326	0.03523	2.36331	0.0183	0.13646	0.06287	2.17028	0.0307	0.05653	0.04259	1.32719	0.1849
128	rs233714_C	-0.0306	0.0419	-0.7299	0.4656	-0.1222	0.07788	-1.5691	0.1176	0.00885	0.04954	0.17856	0.85834

129	rs494273_T	0.08326	0.03523	2.36331	0.0183	0.13646	0.06287	2.17028	0.0307	0.05653	0.04259	1.32719	0.1849
130	rs186763438_C	-0.0508	0.10242	-0.4961	0.61994	-0.0774	0.21153	-0.3659	0.71464	-0.0399	0.11634	-0.3428	0.73186
131	rs491825_T	0.08055	0.03706	2.17369	0.02996	0.1815	0.06821	2.66081	0.00818	0.03161	0.04398	0.71862	0.47263
132	rs797611_T	0.07853	0.03702	2.12137	0.03414	0.17356	0.06779	2.56012	0.01091	0.03219	0.04407	0.7305	0.46534
133	rs170867_A	-0.0204	0.04658	-0.4376	0.66176	-0.1356	0.08557	-1.585	0.11394	0.03046	0.05534	0.55048	0.58217
134	rs233713_A	-0.1468	0.08663	-1.6948	0.09042	-0.3337	0.15857	-2.1045	0.03609	-0.0523	0.10317	-0.5073	0.61212
135	rs35786178_A	0.02342	0.08036	0.29141	0.7708	0.0373	0.14595	0.25553	0.79847	0.02443	0.09626	0.25382	0.79971
136	rs233712_G	-0.024	0.04449	-0.54	0.58932	-0.1194	0.08159	-1.4641	0.14414	0.01797	0.05292	0.33953	0.73432
137	rs233711_A	-0.024	0.04449	-0.54	0.58932	-0.1194	0.08159	-1.4641	0.14414	0.01797	0.05292	0.33953	0.73432
138	rs51357_A	-0.0257	0.04447	-0.5786	0.56298	-0.1234	0.08146	-1.5152	0.13068	0.01797	0.05292	0.33953	0.73432
139	rs7971590_G	-0.0208	0.04639	-0.4473	0.65476	-0.1378	0.0843	-1.6342	0.10319	0.03365	0.05538	0.60757	0.54368
140	rs10850066_A	-0.0208	0.04639	-0.4473	0.65476	-0.1378	0.0843	-1.6342	0.10319	0.03365	0.05538	0.60757	0.54368
141	rs12297526_A	-0.0389	0.04356	-0.892	0.37258	-0.1217	0.08013	-1.5182	0.12993	-0.0033	0.05179	-0.0639	0.94903
142	rs7971951_G	-0.0238	0.04449	-0.5355	0.59244	-0.1234	0.08146	-1.5152	0.13068	0.02085	0.05295	0.39385	0.69381
143	rs10850067_T	0.01614	0.04826	0.33449	0.73808	-0.0054	0.0859	-0.0625	0.95021	0.03075	0.05841	0.52653	0.5987
144	rs117746508_A	0.18035	0.07509	2.4019	0.01649	-0.0523	0.13145	-0.3982	0.69074	0.3088	0.09123	3.38474	0.00075
145	rs10492017_A	0.06794	0.04237	1.60367	0.1091	0.24371	0.0778	3.13234	0.00189	-0.0097	0.05016	-0.1929	0.84707
146	rs6489850_A	-0.0221	0.04451	-0.4968	0.61942	-0.1194	0.08159	-1.4641	0.14414	0.02085	0.05295	0.39385	0.69381
147	rs4766986_T	-0.1113	0.08658	-1.2858	0.1988	-0.2881	0.15639	-1.8422	0.06636	-0.0188	0.10382	-0.1808	0.85658
148	rs4766987_T	-0.021	0.04639	-0.452	0.65136	-0.1336	0.08447	-1.5819	0.11464	0.03046	0.05534	0.55048	0.58217
149	rs77063424_T	-0.0482	0.14187	-0.3395	0.7343	0.05301	0.26463	0.2003	0.84137	-0.0993	0.1677	-0.5919	0.55411
150	rs138360192_T	-0.0482	0.14187	-0.3395	0.7343	0.05301	0.26463	0.2003	0.84137	-0.0993	0.1677	-0.5919	0.55411
151	rs104756_G	-0.021	0.04639	-0.452	0.65136	-0.1336	0.08447	-1.5819	0.11464	0.03046	0.05534	0.55048	0.58217
152	rs232917_G	-0.024	0.04449	-0.54	0.58932	-0.1194	0.08159	-1.4641	0.14414	0.01797	0.05292	0.33953	0.73432
153	rs79366294_C	-0.0482	0.14187	-0.3395	0.7343	0.05301	0.26463	0.2003	0.84137	-0.0993	0.1677	-0.5919	0.55411
154	rs232919_C	-0.0181	0.04639	-0.39	0.69663	-0.1336	0.08447	-1.5819	0.11464	0.03461	0.05533	0.62549	0.53186
155	rs73194705_G	0.03815	0.06134	0.62192	0.53413	-0.0563	0.10605	-0.5308	0.59589	0.10182	0.07561	1.34663	0.17856
156	rs61942312_C	0.07509	0.03712	2.02279	0.04336	0.17469	0.06775	2.57833	0.01036	0.02653	0.04426	0.59933	0.54916
157	rs11615816_T	-0.0482	0.14187	-0.3395	0.7343	0.05301	0.26463	0.2003	0.84137	-0.0993	0.1677	-0.5919	0.55411
158	rs11615820_T	-0.0482	0.14187	-0.3395	0.7343	0.05301	0.26463	0.2003	0.84137	-0.0993	0.1677	-0.5919	0.55411
159	rs232920_C	-0.0194	0.04445	-0.4364	0.66261	-0.1194	0.08159	-1.4641	0.14414	0.02449	0.05288	0.46318	0.64339
160	rs61942313_C	0.08036	0.12126	0.66272	0.50766	-0.0152	0.23363	-0.0652	0.94805	0.12652	0.14132	0.89529	0.37096
161	rs76356460_C	-0.0482	0.14187	-0.3395	0.7343	0.05301	0.26463	0.2003	0.84137	-0.0993	0.1677	-0.5919	0.55411
162	rs232922_T	-0.059	0.04447	-1.326	0.18513	-0.1151	0.07918	-1.4534	0.14708	-0.033	0.0537	-0.6142	0.53926
163	rs61942314_T	0.07577	0.03713	2.04054	0.04156	0.16592	0.06783	2.4461	0.01497	0.03157	0.04425	0.7134	0.47585
164	rs11611483_G	-0.0482	0.14187	-0.3395	0.7343	0.05301	0.26463	0.2003	0.84137	-0.0993	0.1677	-0.5919	0.55411
165	rs75479266_A	-0.0478	0.1032	-0.4631	0.64336	-0.0774	0.21153	-0.3659	0.71464	-0.0348	0.11753	-0.2965	0.76697
166	rs79754842_A	-0.0482	0.14187	-0.3395	0.7343	0.05301	0.26463	0.2003	0.84137	-0.0993	0.1677	-0.5919	0.55411
167	rs75448466_A	-0.0482	0.14187	-0.3395	0.7343	0.05301	0.26463	0.2003	0.84137	-0.0993	0.1677	-0.5919	0.55411
168	rs117784192_G	-0.0482	0.14187	-0.3395	0.7343	0.05301	0.26463	0.2003	0.84137	-0.0993	0.1677	-0.5919	0.55411
169	rs73194711_T	0.0776	0.1226	0.63294	0.52692	-0.0152	0.23363	-0.0652	0.94805	0.12488	0.14357	0.86984	0.3847
170	rs17824620_A	0.06196	0.03743	1.65552	0.09813	0.14278	0.06908	2.06679	0.03954	0.02353	0.04441	0.52986	0.59639
171	rs12423966_T	-0.0302	0.14413	-0.2096	0.83405	0.12558	0.27875	0.45052	0.65264	-0.0993	0.1677	-0.5919	0.55411
172	rs232923_C	0.01807	0.03499	0.51635	0.60572	0.05519	0.06428	0.8585	0.39124	-0.0035	0.04168	-0.0839	0.93318
173	rs16942146_A	-0.0302	0.14413	-0.2096	0.83405	0.12558	0.27875	0.45052	0.65264	-0.0993	0.1677	-0.5919	0.55411
174	rs232924_C	-0.0069	0.03523	-0.1951	0.84539	-0.0502	0.06459	-0.7775	0.43741	0.01772	0.04206	0.4213	0.67367
175	rs4766988_A	0.06361	0.0376	1.69178	0.091	0.14015	0.06898	2.03177	0.04298	0.02608	0.04476	0.58263	0.56034
176	rs7314882_T	-0.0095	0.14176	-0.0669	0.94669	0.12558	0.27875	0.45052	0.65264	-0.0684	0.16409	-0.4171	0.67677
177	rs232925_C	0.01546	0.03495	0.44239	0.65831	0.04916	0.06387	0.76971	0.44203	-0.0043	0.04173	-0.1033	0.91775
178	rs232926_G	-0.0559	0.04405	-1.2699	0.20441	-0.098	0.07764	-1.2621	0.20783	-0.0375	0.0535	-0.7012	0.48339
179	rs232927_A	-0.0115	0.03561	-0.3237	0.74623	-0.0691	0.06574	-1.0517	0.29371	0.0163	0.04236	0.38474	0.70055
180	rs232929_T	-0.065	0.04553	-1.4285	0.15345	-0.0675	0.08154	-0.8278	0.40836	-0.0694	0.05489	-1.2644	0.20654
181	rs76384738_C	0.12213	0.09288	1.31502	0.18881	0.23409	0.17729	1.32035	0.18764	0.07542	0.10893	0.69238	0.48894
182	rs151162036_T	-0.0607	0.13249	-0.4579	0.64713	0.12176	0.24243	0.50224	0.61583	-0.1536	0.15791	-0.9725	0.33116
183	rs232933_G	-0.0015	0.03451	-0.0442	0.96473	0.04089	0.06183	0.66134	0.50886	-0.0283	0.04164	-0.6798	0.49685
184	rs232934_G	-0.0816	0.0506	-1.612	0.10727	-0.1589	0.09175	-1.7314	0.08433	-0.0513	0.0606	-0.8458	0.39795
185	rs232935_T	-0.0117	0.03469	-0.3362	0.73683	0.03889	0.06272	0.61996	0.53571	-0.0426	0.04168	-1.0209	0.30766
186	rs4767948_A	0.04425	0.03888	1.13836	0.25524	0.12976	0.06996	1.85468	0.06454	0.00012	0.04662	0.00248	0.99802
187	rs7133166_T	0.0491	0.03878	1.26612	0.20577	0.12976	0.06996	1.85468	0.06454	0.00756	0.04646	0.16275	0.87077
188	rs74237650_T	0.04748	0.03878	1.22431	0.22112	0.12976	0.06996	1.85468	0.06454	0.00514	0.04645	0.11057	0.91199
189	rs74372924_T	0.04762	0.03878	1.2279	0.21977	0.12976	0.06996	1.85468	0.06454	0.00543	0.04646	0.11688	0.90699
190	rs79113984_A	0.04762	0.03878	1.2279	0.21977	0.12976	0.06996	1.85468	0.06454	0.00543	0.04646	0.11688	0.90699
191	rs232936_T	-0.0849	0.0478	-1.7761	0.07602	-0.1215	0.08601	-1.4128	0.15867	-0.0736	0.05748	-1.2808	0.20071
192	rs232937_T	-0.0849	0.0478	-1.7761	0.07602	-0.1215	0.08601	-1.4128	0.15867	-0.0736	0.05748	-1.2808	0.20071
193	rs4766648_A	0.04762	0.03878	1.2279	0.21977	0.12976	0.06996	1.85468	0.06454	0.00543	0.04646	0.11688	0.90699
194	rs77885072_C	0.04762	0.03878	1.2279	0.21977	0.12976	0.06996	1.85468	0.06454	0.00543	0.04646	0.11688	0.90699
195	rs117776523_C	-0.0589	0.09872	-0.5964	0.55105	-0.0823	0.21117	-0.3899	0.69685	-0.0485	0.1108	-0.4375	0.6619

196	rs56278638_T	0.04872	0.03878	1.25642	0.20926	0.12976	0.06996	1.85468	0.06454	0.00716	0.04645	0.15423	0.87747
197	rs2158241_A	0.04872	0.03878	1.25642	0.20926	0.12976	0.06996	1.85468	0.06454	0.00716	0.04645	0.15423	0.87747
198	rs1344358_T	0.04872	0.03878	1.25642	0.20926	0.12976	0.06996	1.85468	0.06454	0.00716	0.04645	0.15423	0.87747
199	rs379808_T	-0.0879	0.04735	-1.8561	0.06373	-0.1261	0.08579	-1.4703	0.14245	-0.0762	0.05676	-1.3429	0.17976
200	rs11066383_A	-0.0879	0.04735	-1.8561	0.06373	-0.1261	0.08579	-1.4703	0.14245	-0.0762	0.05676	-1.3429	0.17976
201	rs1015249_T	0.0513	0.03868	1.32619	0.18508	0.12976	0.06996	1.85468	0.06454	0.01106	0.0463	0.23883	0.81131
202	rs78550051_A	-0.0374	0.0727	-0.5147	0.60686	-0.1138	0.11777	-0.966	0.33473	-0.0059	0.09387	-0.0629	0.94986
203	rs10492018_A	0.05235	0.03868	1.3535	0.1762	0.12976	0.06996	1.85468	0.06454	0.01231	0.0463	0.26586	0.79043
204	rs73196540_C	-0.0853	0.04768	-1.789	0.07392	-0.1215	0.08601	-1.4128	0.15867	-0.0749	0.0573	-1.307	0.19165
205	rs6489852_T	-0.0988	0.04653	-2.1225	0.03404	-0.125	0.08502	-1.4697	0.14259	-0.0945	0.0556	-1.7002	0.08956
206	rs7301951_G	-0.089	0.05039	-1.7653	0.07782	-0.156	0.09167	-1.7013	0.08984	-0.0639	0.06023	-1.0612	0.28896
207	rs6489853_C	-0.0988	0.04645	-2.1274	0.03363	-0.125	0.08502	-1.4697	0.14259	-0.0948	0.05546	-1.7098	0.08776

Supp. Table S6
PTPN11 gx ~ CD33 SNP + PC1 + PC2 + PC3
Test for the effect of CD33 SNP on PTPN11 gx

		All				stratified by male				stratified by female			
index	SNP	b	se	t	p	b	se	t	p	b	se	t	p
1	rs117613660_A	0.028	0.0368	0.7619	0.4463	-0.0151	0.0629	-0.241	0.8097	0.0462	0.0453	1.02	0.3081
2	rs3865444_A	-0.0178	0.0113	-1.5699	0.1167	-0.0152	0.0181	-0.8392	0.402	-0.0184	0.0145	-1.2684	0.2051
3	rs2459141_C	0.0128	0.0108	1.1865	0.2357	-0.0022	0.0173	-0.1283	0.898	0.0204	0.0138	1.4805	0.1392
4	rs117255828_T	0.028	0.0368	0.7619	0.4463	-0.0151	0.0629	-0.241	0.8097	0.0462	0.0453	1.02	0.3081
5	rs12459419_T	-0.0177	0.0113	-1.5639	0.1181	-0.0146	0.0181	-0.8079	0.4197	-0.0185	0.0145	-1.2749	0.2028
6	rs2455069_G	0.0136	0.0108	1.2576	0.2088	-0.0007	0.0174	-0.0382	0.9696	0.0204	0.0137	1.4842	0.1382
7	rs7245846_A	-0.0142	0.0111	-1.2739	0.203	-0.0171	0.0177	-0.9677	0.3339	-0.0117	0.0142	-0.8214	0.4117
8	rs74796228_A	0.028	0.0368	0.7619	0.4463	-0.0151	0.0629	-0.241	0.8097	0.0462	0.0453	1.02	0.3081
9	rs33978622_C	-0.0179	0.0112	-1.5917	0.1118	-0.0188	0.0179	-1.0483	0.2953	-0.0166	0.0143	-1.1566	0.2478
10	rs73932887_A	0.0269	0.0364	0.7388	0.4602	-0.0151	0.0629	-0.241	0.8097	0.0441	0.0446	0.989	0.323
11	rs34813869_G	-0.0145	0.0111	-1.303	0.1929	-0.0189	0.0177	-1.0634	0.2884	-0.0113	0.0142	-0.7964	0.4261
12	rs73932888_C	0.0247	0.0363	0.6804	0.4964	-0.0151	0.0629	-0.241	0.8097	0.0415	0.0446	0.9312	0.3521
13	rs1354106_G	-0.0144	0.0111	-1.3017	0.1933	-0.0189	0.0177	-1.0634	0.2884	-0.0112	0.0141	-0.7944	0.4273
14	rs35112940_A	-0.0165	0.0129	-1.2715	0.2039	-0.0136	0.0201	-0.6752	0.5	-0.0189	0.0167	-1.1317	0.2582
15	rs10409348_A	0.015	0.0107	1.4035	0.1608	0.0287	0.0179	1.5973	0.1112	0.0082	0.0132	0.6232	0.5334
16	rs1399839_C	-0.0151	0.0102	-1.475	0.1405	-0.0198	0.0165	-1.2053	0.229	-0.0133	0.013	-1.0172	0.3094
17	rs273653_A	-0.0153	0.0102	-1.4922	0.136	-0.0189	0.0164	-1.1491	0.2513	-0.014	0.0131	-1.0695	0.2852
18	rs78347527_A	-0.0119	0.0335	-0.355	0.7226	-0.0092	0.0631	-0.1459	0.8841	-0.0121	0.0399	-0.3037	0.7614
19	rs273652_C	0.0108	0.0105	1.0323	0.3022	0.0165	0.0174	0.9527	0.3415	0.0088	0.0131	0.6708	0.5026
20	rs1803254_C	0.0217	0.0214	1.0137	0.3109	0.0202	0.0367	0.5505	0.5823	0.0216	0.0264	0.8186	0.4133
21	rs8103085_T	0.0217	0.0214	1.0137	0.3109	0.0202	0.0367	0.5505	0.5823	0.0216	0.0264	0.8186	0.4133
22	rs1697531_G	0.0101	0.0105	0.9642	0.3352	0.0165	0.0174	0.9527	0.3415	0.0077	0.0131	0.5908	0.5548
23	rs1034521_G	0.0249	0.0207	1.2049	0.2285	0.0202	0.0367	0.5505	0.5823	0.0261	0.0252	1.0347	0.3012
24	rs12461354_A	0.0249	0.0207	1.2049	0.2285	0.0202	0.0367	0.5505	0.5823	0.0261	0.0252	1.0347	0.3012
25	rs76248157_T	0.0249	0.0207	1.2049	0.2285	0.0202	0.0367	0.5505	0.5823	0.0261	0.0252	1.0347	0.3012
26	rs73612300_C	0.0249	0.0207	1.2049	0.2285	0.0202	0.0367	0.5505	0.5823	0.0261	0.0252	1.0347	0.3012
27	rs273651_A	0.0149	0.0102	1.4555	0.1459	0.0189	0.0164	1.1491	0.2513	0.0134	0.013	1.0262	0.3052
28	rs169275_T	0.0092	0.0105	0.8804	0.3788	0.0165	0.0174	0.9527	0.3415	0.0065	0.0131	0.4947	0.621
29	rs273650_A	0.0149	0.0102	1.4555	0.1459	0.0189	0.0164	1.1491	0.2513	0.0134	0.013	1.0262	0.3052
30	rs1566578_T	0.0249	0.0207	1.2049	0.2285	0.0202	0.0367	0.5505	0.5823	0.0261	0.0252	1.0347	0.3012
31	rs145323276_C	0.014	0.0224	0.6284	0.5299	-0.0035	0.0412	-0.0847	0.9326	0.0206	0.0268	0.7668	0.4435
32	rs11084061_A	0.0249	0.0207	1.2049	0.2285	0.0202	0.0367	0.5505	0.5823	0.0261	0.0252	1.0347	0.3012
33	rs273649_C	0.0092	0.0105	0.8804	0.3788	0.0165	0.0174	0.9527	0.3415	0.0065	0.0131	0.4947	0.621
34	rs11084062_C	0.0249	0.0207	1.2049	0.2285	0.0202	0.0367	0.5505	0.5823	0.0261	0.0252	1.0347	0.3012
35	rs12461282_C	0.0249	0.0207	1.2049	0.2285	0.0202	0.0367	0.5505	0.5823	0.0261	0.0252	1.0347	0.3012
36	rs273648_A	0.0092	0.0105	0.8804	0.3788	0.0165	0.0174	0.9527	0.3415	0.0065	0.0131	0.4947	0.621
37	rs11084063_T	0.0239	0.0207	1.1543	0.2487	0.0202	0.0367	0.5505	0.5823	0.0248	0.0253	0.9804	0.3273
38	rs989504_T	0.0239	0.0207	1.1543	0.2487	0.0202	0.0367	0.5505	0.5823	0.0248	0.0253	0.9804	0.3273
39	rs273647_C	0.0149	0.0102	1.4555	0.1459	0.0189	0.0164	1.1491	0.2513	0.0134	0.013	1.0262	0.3052
40	rs989502_A	0.0249	0.0207	1.2049	0.2285	0.0202	0.0367	0.5505	0.5823	0.0261	0.0252	1.0347	0.3012
41	rs989505_A	0.0249	0.0207	1.2049	0.2285	0.0202	0.0367	0.5505	0.5823	0.0261	0.0252	1.0347	0.3012
42	rs1319675_A	0.0249	0.0207	1.2049	0.2285	0.0202	0.0367	0.5505	0.5823	0.0261	0.0252	1.0347	0.3012
43	rs273646_T	0.0092	0.0105	0.8804	0.3788	0.0165	0.0174	0.9527	0.3415	0.0065	0.0131	0.4947	0.621
44	rs273645_T	0.0092	0.0105	0.8804	0.3788	0.0165	0.0174	0.9527	0.3415	0.0065	0.0131	0.4947	0.621
45	rs7253829_C	0.0249	0.0207	1.2049	0.2285	0.0202	0.0367	0.5505	0.5823	0.0261	0.0252	1.0347	0.3012
46	rs7253831_A	0.0249	0.0207	1.2049	0.2285	0.0202	0.0367	0.5505	0.5823	0.0261	0.0252	1.0347	0.3012
47	rs17801843_T	0.0249	0.0207	1.2049	0.2285	0.0202	0.0367	0.5505	0.5823	0.0261	0.0252	1.0347	0.3012
48	rs273644_A	0.01	0.0105	0.9577	0.3385	0.018	0.0174	1.0312	0.3032	0.007	0.0131	0.5355	0.5925
49	rs273643_T	0.0108	0.0105	1.0205	0.3077	0.0212	0.0175	1.2076	0.2281	0.0067	0.0132	0.5115	0.6092
50	rs273642_C	-0.0167	0.0103	-1.6262	0.1042	-0.023	0.0166	-1.3908	0.1652	-0.0141	0.0131	-1.0827	0.2793
51	rs273641_C	0.0108	0.0105	1.0205	0.3077	0.0212	0.0175	1.2076	0.2281	0.0067	0.0132	0.5115	0.6092
52	rs7256372_T	0.0241	0.0206	1.1693	0.2426	0.0202	0.0367	0.5505	0.5823	0.0252	0.0251	1.0006	0.3174
53	rs71358823_G	-0.006	0.0261	-0.2304	0.8179	0.009	0.0452	0.1995	0.842	-0.0132	0.0321	-0.4123	0.6803
54	rs1501447_G	0.0241	0.0206	1.1693	0.2426	0.0202	0.0367	0.5505	0.5823	0.0252	0.0251	1.0006	0.3174
55	rs1501448_T	0.0241	0.0206	1.1693	0.2426	0.0202	0.0367	0.5505	0.5823	0.0252	0.0251	1.0006	0.3174
56	rs166653_G	0.0102	0.0105	0.9637	0.3354	0.0212	0.0175	1.2076	0.2281	0.0059	0.0132	0.4465	0.6554
57	rs273619_T	0.0102	0.0105	0.9637	0.3354	0.0212	0.0175	1.2076	0.2281	0.0059	0.0132	0.4465	0.6554

Supp. Table S7. Causal Mediation Analysis. Test whether PTPN6 gx mediates the association between CD33 gx and a trait
Model: CD33 gx > PTPN6gx (mediator) > trait
Gene expression (gx) in ROSMAP DLPFC was used

trait	Effect	b	CI.L	CI.U	p
amyloid	Average Casaul Mediation Effect (ACME)	0.019	-0.054	0.086	0.638
amyloid	Average Direct Effect (ADE)	0.066	-0.043	0.180	0.238
amyloid	Total Effect	0.085	0.000	0.176	0.052
amyloid	Proportion Mediated (PM)	0.221	-1.268	2.526	0.654
tangles	Average Casaul Mediation Effect (ACME)	-0.008	-0.086	0.071	0.824
tangles	Average Direct Effect (ADE)	0.074	-0.064	0.208	0.310
tangles	Total Effect	0.066	-0.040	0.172	0.218
tangles	Proportion Mediated (PM)	-0.115	-4.297	4.206	0.818
pathologic AD	Average Casaul Mediation Effect (ACME)	0.007	-0.023	0.038	0.682
pathologic AD	Average Direct Effect (ADE)	0.022	-0.026	0.068	0.400
pathologic AD	Total Effect	0.030	-0.009	0.064	0.150
pathologic AD	Proportion Mediated (PM)	0.242	-3.080	3.550	0.736
AD dementia	Average Casaul Mediation Effect (ACME)	-0.020	-0.053	0.018	0.272
AD dementia	Average Direct Effect (ADE)	0.072	0.013	0.136	0.018
AD dementia	Total Effect	0.052	0.004	0.100	0.032
AD dementia	Proportion Mediated (PM)	-0.389	-3.050	0.627	0.300
cognitive decline	Average Casaul Mediation Effect (ACME)	0.001	-0.005	0.007	0.866
cognitive decline	Average Direct Effect (ADE)	-0.007	-0.017	0.001	0.114
cognitive decline	Total Effect	-0.007	-0.014	0.000	0.058
cognitive decline	Proportion Mediated (PM)	-0.075	-2.525	1.507	0.864
tdp43	Average Casaul Mediation Effect (ACME)	-0.007	-0.038	0.025	0.660
tdp43	Average Direct Effect (ADE)	0.040	-0.011	0.098	0.136
tdp43	Total Effect	0.033	-0.003	0.076	0.082
tdp43	Proportion Mediated (PM)	-0.215	-3.013	2.195	0.686
hippocampal sclerosis	Average Casaul Mediation Effect (ACME)	-0.012	-0.040	0.009	0.276
hippocampal sclerosis	Average Direct Effect (ADE)	0.026	-0.005	0.058	0.116
hippocampal sclerosis	Total Effect	0.016	-0.007	0.045	0.176
hippocampal sclerosis	Proportion Mediated (PM)	-0.734	-5.973	3.243	0.376
Global AD pathology burden	Average Casaul Mediation Effect (ACME)	0.020	-0.017	0.055	0.290
Global AD pathology burden	Average Direct Effect (ADE)	0.018	-0.038	0.081	0.538
Global AD pathology burden	Total Effect	0.038	-0.006	0.088	0.090
Global AD pathology burden	Proportion Mediated (PM)	0.534	-3.249	5.311	0.360

Supp. Table S8. Causal Mediation Analysis. Test whether CD33 gx mediates the association between PTPN6 gx and trait
Model: PTPN6 gx > CD33 gx (mediator) > trait
Gene expression (gx) in ROSMAP DLPFC was used

trait	Effect	b	CI.L	CI.U	p
amyloid	Average Casaul Mediation Effect (ACME)	0.054	-0.042	0.150	0.270
amyloid	Average Direct Effect (ADE)	0.038	-0.116	0.193	0.614
amyloid	Total Effect	0.093	-0.019	0.212	0.112
amyloid	Proportion Mediated (PM)	0.585	-4.010	5.039	0.362
tangles	Average Casaul Mediation Effect (ACME)	0.060	-0.041	0.171	0.248
tangles	Average Direct Effect (ADE)	-0.016	-0.177	0.137	0.810
tangles	Total Effect	0.044	-0.084	0.172	0.514
tangles	Proportion Mediated (PM)	1.354	-16.007	10.850	0.606
pathologic AD	Average Casaul Mediation Effect (ACME)	0.018	-0.018	0.058	0.372
pathologic AD	Average Direct Effect (ADE)	0.015	-0.043	0.072	0.620
pathologic AD	Total Effect	0.033	-0.012	0.079	0.176
pathologic AD	Proportion Mediated (PM)	0.542	-5.124	4.996	0.508
AD dementia	Average Casaul Mediation Effect (ACME)	0.055	0.012	0.100	**0.008**
AD dementia	Average Direct Effect (ADE)	-0.042	-0.119	0.040	0.278
AD dementia	Total Effect	0.014	-0.045	0.070	0.666
AD dementia	Proportion Mediated (PM)	4.092	-22.768	27.120	0.670
cognitive decline	Average Casaul Mediation Effect (ACME)	-0.006	-0.014	0.001	0.128
cognitive decline	Average Direct Effect (ADE)	0.001	-0.011	0.014	0.844
cognitive decline	Total Effect	-0.005	-0.013	0.005	0.320
cognitive decline	Proportion Mediated (PM)	1.222	-6.751	12.854	0.408
tdp43	Average Casaul Mediation Effect (ACME)	0.031	-0.006	0.068	0.104
tdp43	Average Direct Effect (ADE)	-0.015	-0.077	0.046	0.610
tdp43	Total Effect	0.017	-0.029	0.070	0.510
tdp43	Proportion Mediated (PM)	1.818	-16.781	14.761	0.574
hippocampal sclerosis	Average Casaul Mediation Effect (ACME)	0.018	-0.004	0.039	0.122
hippocampal sclerosis	Average Direct Effect (ADE)	-0.023	-0.067	0.023	0.290
hippocampal sclerosis	Total Effect	0.000	-0.023	0.031	0.988
hippocampal sclerosis	Proportion Mediated (PM)	-167.325	-20.360	28.083	0.986
Global AD pathology burden	Average Casaul Mediation Effect (ACME)	0.014	-0.036	0.062	0.576
Global AD pathology burden	Average Direct Effect (ADE)	0.042	-0.035	0.117	0.294
Global AD pathology burden	Total Effect	0.056	-0.001	0.111	0.062
Global AD pathology burden	Proportion Mediated (PM)	0.256	-1.435	3.749	0.582

Supp. Table S9
Causal Mediation Analysis
CD33 gx > PTPN11gx (mediator) > trait
Test whether PTPN11 gx mediates the association between CD33 gx and trait
Gene expression (gx) in ROSMAP DLPFC n=1092 were used

	trait	b	CI.L	CI.U	p
ACME	amyloid	-0.0099235	-0.0240679	-0.0005356	0.038
ACME	tangles	-0.0104871	-0.0241672	-5.21E-05	0.048
ACME	pathologic AD	-0.0033107	-0.0085403	-0.0001354	0.038
ACME	AD dementia	-0.0056434	-0.0136514	-2.60E-05	0.05
ACME	cognitive decline	0.00056379	-5.78E-05	0.00147886	0.076
ACME	tdp43	0.00101763	-0.0016517	0.00399363	0.466
ACME	hippocampal sclerosis	-0.0005352	-0.0032079	0.00182156	0.656
ACME	Global AD pathology burden	-0.0039425	-0.0102861	-0.0001363	0.042

Supp. Table S10
Causal Mediation Analysis
PTPN11 gx > CD33 gx (mediator) > trait
Test whether CD33 gx mediates the association between PTPN11 gx and trait
Gene expression (gx) in ROSMAP DLPFC n=1092 were used

	trait	b	CI.L	CI.U	p
ACME	amyloid	-0.0221043	-0.0618082	0.00050425	0.064
ACME	tangles	-0.0186785	-0.0554894	0.0062796	0.17
ACME	pathologic AD	-0.0065626	-0.0175517	0.00063662	0.086
ACME	AD dementia	-0.0126198	-0.0330499	0.0007686	0.072
ACME	cognitive decline	0.00154916	-0.0002034	0.00433502	0.102
ACME	tdp43	-0.0057666	-0.0179219	0.0014328	0.158
ACME	hippocampal sclerosis	-0.0045877	-0.0171835	0.00182583	0.18
ACME	Global AD pathology burden	-0.0105944	-0.0297339	0.00101966	0.068

1. Vonsattel JP, Del Amaya MP, Keller CE. Twenty-first century brain banking. Processing brains for research: the Columbia University methods. *Acta Neuropathol*. May 2008;115(5):509-32. doi:10.1007/s00401-007-0311-9
2. Chatila ZK, Yadav A, Mares J, et al. RNA- and ATAC-sequencing Reveals a Unique CD83+ Microglial Population Focally Depleted in Parkinson's Disease. *bioRxiv*. May 17 2023;doi:10.1101/2023.05.17.540842
3. Ryan KJ, White CC, Patel K, et al. A human microglia-like cellular model for assessing the effects of neurodegenerative disease gene variants. *Sci Transl Med*. Dec 2017;9(421)doi:10.1126/scitranslmed.aai7635
